# A Factor H-Fc fusion protein increases complement-mediated opsonophagocytosis and killing of community associated methicillin-resistant *Staphylococcus aureus*

**DOI:** 10.1371/journal.pone.0265774

**Published:** 2022-03-24

**Authors:** Megan A. G. Sage, Katelyn D. Cranmer, Michele L. Semeraro, Shelby Ma, Elena V. Galkina, Y. Tran, Keith L. Wycoff, Julia A. Sharp

**Affiliations:** 1 Department of Microbiology and Molecular Cell Biology, Eastern Virginia Medical School, Norfolk, VA, United States of America; 2 Planet Biotechnology, Inc., Hayward, CA, United States of America; Pusan National University, REPUBLIC OF KOREA

## Abstract

*Staphylococcus aureus* employs a multitude of immune-evasive tactics to circumvent host defenses including the complement system, a component of innate immunity central to controlling bacterial infections. With antibiotic resistance becoming increasingly common, there is a dire need for novel therapies. Previously, we have shown that *S*. *aureus* binds the complement regulator factor H (FH) via surface protein SdrE to inhibit complement. To address the need for novel therapeutics and take advantage of the FH:SdrE interaction, we examined the effect of a fusion protein comprised of the SdrE-interacting domain of FH coupled with IgG Fc on complement-mediated opsonophagocytosis and bacterial killing of community associated methicillin-resistant *S*. *aureus*. *S*. *aureus* bound significantly more FH-Fc compared to Fc-control proteins and FH-Fc competed with serum FH for *S*. *aureus* binding. FH-Fc treatment increased C3-fragment opsonization of *S*. *aureus* for both C3b and iC3b, and boosted generation of the anaphylatoxin C5a. In 5 and 10% serum, FH-Fc treatment significantly increased *S*. *aureus* killing by polymorphonuclear cells. This anti-staphylococcal effect was evident in 75% (3/4) of clinical isolates tested. This study demonstrates that FH-Fc fusion proteins have the potential to mitigate the protective effects of bound serum FH rendering *S*. *aureus* more vulnerable to the host immune system. Thus, we report the promise of virulence-factor-targeted fusion-proteins as an avenue for prospective anti-staphylococcal therapeutic development.

## Introduction

*Staphylococcus aureus* is a highly effective human pathogen that causes a wide-range of infections including those of the skin and soft tissue, bone, heart and joints [1], resulting in significant morbidity and mortality worldwide. Antibiotic resistance, especially to clindamycin, is increasing for both methicillin-sensitive and methicillin-resistant *S*. *aureus* (MRSA) [2]. To date, all vaccination trials to prevent invasive *S*. *aureus* infections in humans have failed [3]. Thus, novel therapies are in dire need.

As a master of immune evasion, *S*. *aureus* specifically targets the complement system, a critical component of innate immunity comprised of more than 50 soluble and surface-bound proteins. Operating in a catalytic cascade, complement activation results in opsonization of the bacterial surface and anaphylatoxin release to facilitate bacterial uptake and destruction by phagocytes [4–6]. The three complement pathways converge at the formation of C3-convertase, an enzyme central to the generation of the major opsonin C3b. The classical pathway is activated by bound antibody (IgM or multiple IgG) or pentraxins (e.g., C-reactive protein), whereas the lectin pathway becomes activated through the recognition of specific carbohydrate moieties. The alternative pathway is active at a low level (C3-tickover) and readily amplifies all pathways. Recent evidence supports a possible cross-activation between the pathways [7, 8], thereby substantiating the encompassing nature of complement. Due to the potent effects of complement, membrane-bound and soluble complement regulators are critical for protection of host cells and tissues from complement-mediated bystander damage.

*S*. *aureus* produces several soluble proteins to subvert complement-mediated opsonization, including the extracellular complement-binding protein (Ecb), the extracellular fibrinogen-binding protein (Efb), and the staphylococcal complement inhibitor (SCIN). These virulence factors target the formation or function of C3-convertase, thereby affecting opsonization and downstream effects [5, 9, 10]. *S*. *aureus* protein A, a well-known immune-evasive protein, binds the Fc region of immunoglobulin thereby providing a two-pronged inhibitory function to effectively impede C1q-mediated activation of the classical pathway of complement and block FcγR-mediated phagocytosis [1].

To further dampen complement attack, *S*. *aureus* takes advantage of host-protective mechanisms by binding complement regulators including factor H (FH), the major fluid-phase regulator of the alternative pathway. FH downregulates complement by disrupting the alternative pathway C3 convertase and provides co-factor functionality for factor I inactivation of C3b to iC3b. FH contains 20 complement control protein modules (CCPs) with CCP1-4, CCP6-8 and CCP18-20 critical for functions such as complement regulation, self-identification, and binding to C3b (reviewed in [11]). FH simultaneously binds C3 fragments and select glycosaminoglycans on host cells through CCP19-20 to limit further complement activation [12, 13]. Previously, we identified SdrE (serine aspartic acid repeat protein E) as the *S*. *aureus* surface protein that binds FH [14]. SdrE recruits FH by interacting with CCP19-20 [15] to downregulate complement leading to a survival advantage [14].

To take advantage of SdrE:FH binding, we tested fusion proteins comprising CCP18-20, fused to the Fc region of IgG. Binding to *S*. *aureus* by the FH region exposes the IgG Fc components capable of interacting with C1q (the initiating protein of the classical pathway of complement) or with FcγR on phagocytes. Previous studies have demonstrated that similar FH-Fc fusion constructs are efficacious against *Neisseria meningitidis* [16], nontypeable *Haemophilus influenzae* [12], *N*. *gonorrhoeae* [17] and group A streptococci [18], in both *in vitro* and animal infection models.

In this study, we demonstrate that FH-Fc competes with serum FH for binding to *S*. *aureus*, leading to increased complement activation through greater C3-fragment deposition and C5a generation. When challenged with phagocytes, FH-Fc treatment causes a significant increase in complement-mediated opsonophagocytosis of *S*. *aureus* and a reduction in bacterial survival.

## Materials and methods

### Ethics statement

Human blood was obtained from healthy volunteers for generating serum or purifying neutrophils in accordance with IRB 18-05-EX-0109, approved by Eastern Virginia Medical School IRB. Written informed consent was provided by all study participants.

### Buffers

HBS^++^ (HEPES-Buffered Saline): 0.01 M HEPES, 0.15 M NaCl, 135 μM CaCl_2_, 1 mM MgCl_2_, pH 7.4; HBS^++^ was prepared as previously described [19]. The addition of Ca^2+^ and Mg^2+^ permits activation of all complement pathways. EGTA-HBS^+^: 0.01 M HEPES, 0.15 M NaCl, 5 mM MgCl_2_, 8 mM EGTA, pH 7.4. EGTA chelates Ca^2+^ permitting activation of the alternative pathway only. EDTA-HBS: 0.01 M HEPES, 0.15 M NaCl, 0.01 M EDTA, pH 7.4. EDTA chelates the cations Ca^2+^ and Mg^2+^ to inhibit all complement pathways.

### Serum

Normal human serum (NHS) was collected from the blood of at least four healthy human volunteers in accordance with an Institutional Review Board-approved protocol (EVMS IRB 18-05-EX-0109). Serum was extracted from blood as previously described [20], pooled, aliquoted, and stored at -80°C.

### Bacterial strains and growth

*S*. *aureus* strain Reynolds was generously provided by Dr. Kenji Cunnion, EVMS. Strains Newman and Protein A-deficient Newman (*spA* negative) were kindly provided by Dr. Gerald B. Pier, Harvard Medical School. Community associated methicillin resistant *S*. *aureus* (CA-MRSA) clinical isolates were obtained as de-identified, discarded specimens from the Children’s Hospital of the King’s Daughters (CHKD) clinical microbiology laboratory (EVMS IRB 18-5-EX-0109) in Norfolk, VA. Unless otherwise indicated, CA-MRSA isolate R7, a confirmed USA300 strain previously shown to bind FH [6], was the *S*. *aureus* strain studied. Bacteria were grown to mid-log phase (OD_600_ 0.8–1.5) in Columbia broth with 2% NaCl at 37°C, 250 rpm.

### CA-MRSA genotyping

Genomic DNA from CA-MRSA isolates was extracted using heat (99°C for 10 minutes) then examined for sequence type (Multilocus sequence typing (MLST)), staphylococcal cassette chromosome (SCC*mec*) type, and the presence of Panton-Valentine leukocidin (*pvl*) and *sdrE* genes. MLST was performed as previously described [21]. Briefly, gDNA was PCR amplified for seven house-keeping genes; column-purified PCR products were then sequenced by Sanger sequencing methods using the BigDye Terminator v3.1 Cycle Sequencing and Xterminator Purification Kits and the 3130 Genetic Analyzer (Applied Biosystems). Contig sequences were generated with GeneStudio software then queried against the online PubMLST database [22]. SCC*mec* typing, and the presence of *pvl* and *sdrE* genes was determined as described previously [23–26]. Isolates were identified as USA300 based on the following profile: MLST 8, SCC*mec* IVa, and *pvl* positive.

### Fusion-Protein development

Fusion proteins were produced using the pTRAkc plant expression vector, the soil bacterium *Agrobacterium tumefaciens*, and *Nicotiana benthamiana* plants. Plant-derived glycoforms produced by this system have a humanized N-glycan structure [27, 28]. FH-Fc construction, expression and purification was described previously, where FH-Fc and Variant 1 were referenced as strain S2477 and S2493, respectively [17]. The molecular constructs of FH-Fc, FH-Fc variants, and Fc-control proteins used in this study are listed in Table 1; mutations were generated by site-directed mutagenesis. In a previous study, FH-Fc was shown to exert complement-dependent cytotoxicity (CDC) against *N*. *gonorrheae*; similarly, the Fc region of FH-Fc, with a modified linker, was shown to be efficacious at promoting opsonophagocytosis of *N*. *gonorrheae* [17]. Variant 1 (V1) possesses two Fc mutations (D270A/K322A), to abrogate C1q binding [29], and has demonstrated complement inactivity [17]. Variant 2 (V2) contains a codon-optimized sequence encoding the C_H_2 and C_H_3 domains of human IgG3 (GenBank accession no. CAA67886.1), in place of the corresponding domains of IgG1, with the R at position 435 (Eu numbering) replaced with H, conferring both longer *in-vivo* half-life and Protein-A binding (Fc3(435H)). Variant V2R was produced with the relative positions of Fc3 (435H) and FH* reversed (Fc3 at the N-terminus and FH* at the C-terminus). V2R was further modified to introduce three Fc mutations (S267E/H268F/S324T), previously shown to improve binding affinity to C1q and enhance CDC of an anti-CD20 Ab by 23-fold against tumor cells [30], resulting in Variant 5 (V5). The construction of Fc fusion proteins with human dipeptidyl peptidase 4 (DPP4) and decay accelerating factor (DAF) is described elsewhere [31].

**Table 1:** Description of plant-produced FH*-Fc molecules.

Fusion Protein	Binary expression vector name
FH-Fc	pTRAk-c-lph-FH*-(G_4_S)_2_-hFc1
V1 (complement inactive)	pTRAk-c-lph-FH*-(G_4_S)_2_-hFc1(D270A/K322A)
V2	pTRAk-c-lph-FH*-(G_4_S)_2_-hFc3(IgG1 hinge)(435H)
V2R	pTRAk-c-lph-hFc3(IgG1 hinge)(435H)-(G_4_S)_2_-FH*
V5	pTRAk-c-lph-hFc3(IgG1 hinge)(EFT,435H) -(G_4_S)_2_-FH*
DPP4-Fc	pTRAk-c-lph-DPP4(39–766, mV11)-hFc1
DAF-Fc	pTRAk-c-lph-DAF(agly)-Fc(H435A)

FH* denotes FH CCP18-20 with a mutation in CCP19 (D to G at position 1119), which abrogates the ability to bind to C3b-coated host surfaces [32, 33].

### Fluorescent labeling

Fusion proteins FH-Fc and DAF-Fc were fluorescently labeled using a modified protocol for the SiteClick Antibody Azido Modification Kit (Thermo Fisher) using CF Dye BCN 405M (Biotium). FH-Fc and DAF-Fc contain terminal GlcNAc resides only; therefore, no β-galactosidase treatment was applied. Due to the likelihood of protein aggregate formation, labeled proteins were dialyzed in place of spin-column purification.

### Fusion protein binding assays

Bacteria (2.5 × 10^8^ CFU or 1 × 10^8^ CFU) were incubated in PBS with various amounts of fusion protein, for one hour at 37°C as previously described [6]. After washing 3 × with PBS, bound proteins were extracted in 40μL 2% SDS at 95°C for 5 minutes and examined using immunodot-blotting.

### Immunodot-Blotting

Samples were applied to a nitrocellulose membrane using the Bio-Dot Microfiltration Apparatus (Bio-Rad). The membrane was blocked with Odyssey Blocking Buffer (Li-Cor), then probed with goat anti-human IgG CW800 polyclonal antibody (Li-Cor), or goat anti-human FH IgG (CompTech) followed by donkey anti-goat IgG CW800 (Li-Cor). The blot was visualized using the Li-Cor Odyssey CLx Imaging System and the signal intensity of samples was measured via optical densitometry. Translation of signal to quantity was calculated using a standard curve derived from fusion protein standard titrations.

### Flow cytometry

Flow cytometry of human peripheral blood leukocytes was performed as we described before [34]. Briefly, whole blood was collected from healthy volunteers into lithium heparin tubes (BD), centrifuged for 15 min ⊆1000 rpm at 4°C, and plasma removed. The remaining blood was aliquoted (200 μL) then treated with 3mL of ACK lysis buffer (150mM NH_4_Cl, 10mM KHCO_3_, 0.1mM EDTA) for at least 8 min, to lyse red blood cells. After washing twice, leukocytes were incubated with fusion protein (500 ng) for 15 min at 37°C, then washed. Next, cells were incubated with 1μg of Fc block for 15 min at room temperature, followed by staining with anti-CD15 Pacific Blue (clone W6D3, Biolegend) per manufacturer’s instructions, and goat anti-FH IgG (1:400, CompTech). After washing, leukocytes were incubated with FITC-conjugated donkey anti-goat IgG (1:250, Jackson ImmunoResearch Labs) to detect bound fusion protein. Controls for background staining (no fusion protein and secondary probe only) were generated per donor. Cells were fixed with 4% paraformaldehyde then events were collected using Cytek® Aurora Spectral Flow cytometer (Cytek®, USA) and analyzed using SpectroFlow® unmixing software (Cytek®, USA) and FlowJo software (Tree Star, Inc). Neutrophils were identified as FSC^high^/SSC^high^ CD15^+^ cells. Washes and incubations (except lysis) were performed using HBSS without Ca^2+^ and Mg^2+^ (Gibco).

### Fluorescent competition binding assays

CA-MRSA R7 (1 × 10^8^ CFU) was incubated in HBS^++^ with fluorescently labeled FH-Fc or DAF-Fc (62.5 nM) and various amounts of NHS for one hour at 37°C, as previously described [6]. Bacteria were washed thrice, then resuspended in PBS and examined for the presence of bound fusion protein using a SpectraMax iD3 multimode microplate reader (Molecular Devices) at 408 nm excitation/452 nm emission spectra. Fusion-protein molarity was calculated by linear regression of the standard curve generated using a titration of fluorescent fusion protein of known quantity, subtracting background from control wells (bacteria only, NHS only, and/or media only).

### Competition-Binding assays

CA-MRSA R7 (1 × 10^8^ CFU) were incubated in HBS^++^ with 2.5% NHS and various amounts of fusion protein for one hour at 37°C, as previously described [6]. After washing the bacteria 3 × with EDTA-HBS, bound proteins were extracted with 40μL 2% SDS at 95°C for 5 minutes. Protein extracts were examined for FH presence via anti FH-Western Blot and ELISA.

### ELISA

ELISAs for FH, C3, and C5a were performed as previously described, with modifications [6, 14]. Serum FH was detected using mouse anti-human FH CCP1 IgG (Quidel), a region of FH not contained within the fusion protein, followed by HRP-conjugated rabbit anti-mouse IgG (Sigma). Total C3 was detected using chicken anti-human C3 IgY (Millipore), followed by HRP-conjugated goat anti-chicken IgG (Invitrogen); C5a was detected using the DuoSet ELISA Development kit, per manufacturer’s instructions (R&D systems). Plates were developed with TMB substrate, stopped with 2N H_2_SO_4_, and then read at 450nm. Purified FH (CompTech), C3 (CompTech) or C5a (R&D systems) were used as standards for their respective ELISAs.

### Anti-Factor H Western blot

Samples were subjected to reducing SDS-PAGE and transferred to a nitrocellulose membrane (Li-Cor). To reduce non-specific binding of antibody by *S*. *aureus* Fc-binding proteins, DPP4-Fc was added to the initial block. After washing, the membrane was probed with goat anti-FH IgG (CompTech), followed by donkey anti-goat IgG CW800 (Li-Cor), then visualized by Odyssey CLx (Li-Cor). FH bands were assessed using optical densitometry and compared across samples.

### C3-fragment deposition

CA-MRSA R7 was incubated with NHS plus FH-Fc, V1, or no fusion protein in HBS^++^ for 30 minutes at 37°C. Bacteria were washed 3 × with EDTA-HBS to inactivate complement, and C3-fragments were extracted from the bacterial surface with 25mM methylamine, as previously described [6]. The bacteria were centrifuged and the supernatant was examined for C3-fragment presence using C3 ELISA and Western blotting. This assay was also performed in EGTA-HBS^+^, to permit activation of the alternative pathway only.

### C3 Western blot

Samples were subjected to reducing SDS-PAGE, followed by Western-blot analysis using goat anti-C3 IgG (CompTech) and donkey anti-goat IgG CW800 (Li-Cor). Purified C3b and iC3b (CompTech) were used as controls. To measure the relative quantity of C3b and iC3b in samples, we examined the α′ (C3b) and α2 (iC3b) bands by optical densitometry and compared to the no fusion protein samples.

### C5a generation

CA-MRSA R7 (1 × 10^8^ CFU) were incubated with various concentrations of NHS in HBS^++^ with or without FH-Fc or V1, for 15 minutes at 37°C to generate C5a. EDTA was added to a final concentration of 0.8 mM to halt complement; the supernatant was evaluated for C5a presence by ELISA. This assay was also performed in EGTA-HBS^+^, to permit activation of the alternative pathway only.

### Phagocytosis/Killing assay

Whole blood was collected from healthy volunteers into lithium heparin tubes (BD) and polymorphonuclear cells (PMNs) were extracted, using hipaque-ficol density gradient, 3% dextran sedimentation, and hypotonic lysis, as previously described [14]. PMNs were resuspended in cold HBSS without Ca^2+^ or Mg^2+^ (Gibco) to a final concentration of 1 × 10^7^ cells/mL. *S*. *aureus* (2 × 10^7^ CFU) were incubated with various amounts of NHS ± fusion protein in HBSS with Ca^2+^ and Mg^2+^ (Corning), at 37°C for 15 minutes to permit opsonization, in a total volume of 500 μL. PMNs were then added to reaction tubes in a ratio of 1 PMN:10 bacteria. The total volume was increased to 1mL using warm HBSS (with Ca^2+^ and Mg^2+^), and tubes were rotated at 37°C for 75 minutes. Following the incubation, samples were serially diluted in sterile diH_2_O then sterile normal saline, before plating on Columbia agar with 2% NaCl. Following an overnight incubation at 37°C, colonies were counted and percent survival of bacteria (compared to no fusion protein control) was determined.

### Statistical analysis

Statistical significance was calculated by analyzing the data with one- or two-way ANOVA, two-tailed paired Student’s t-test, and competitive binding kinetics using Prism GraphPad 8. *P* values of ≤ 0.05 were considered significant. The Sidak’s multiple comparisons test and the Holm-Sidak method were used, as appropriate.

## Results

### S. aureus binds FH-Fc in a dose-dependent manner

**Fig 1:**
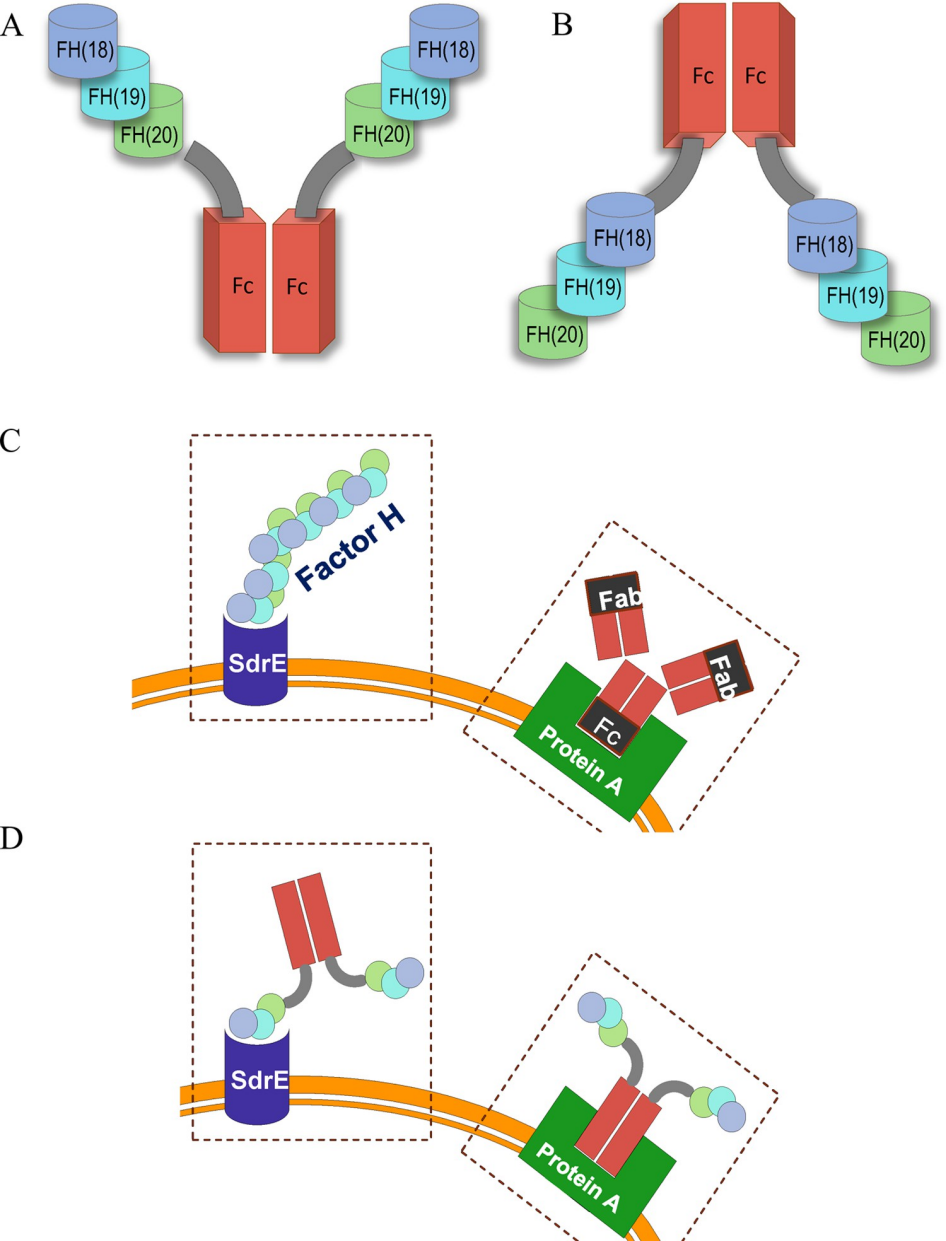
Structure and binding of FH, IgG, and FH-Fc to staphylococcal virulence factors Protein A and SdrE. A, Structure of the homodimeric fusion protein FH-Fc; IgG1 Fc region portrayed as rectangles, FH (CCP18-20) portrayed as cylinders, connected by the IgG1 hinge region. B, Reverse orientation of FH-Fc (Fc at the N-terminus and FH (CCP 18–20) at the C-terminus). C, C-terminus of FH bound by SdrE and Fc domain of IgG bound by Protein A. D, Schematic of FH-Fc fusion protein binding to SdrE and protein A.

**Fig 2:**
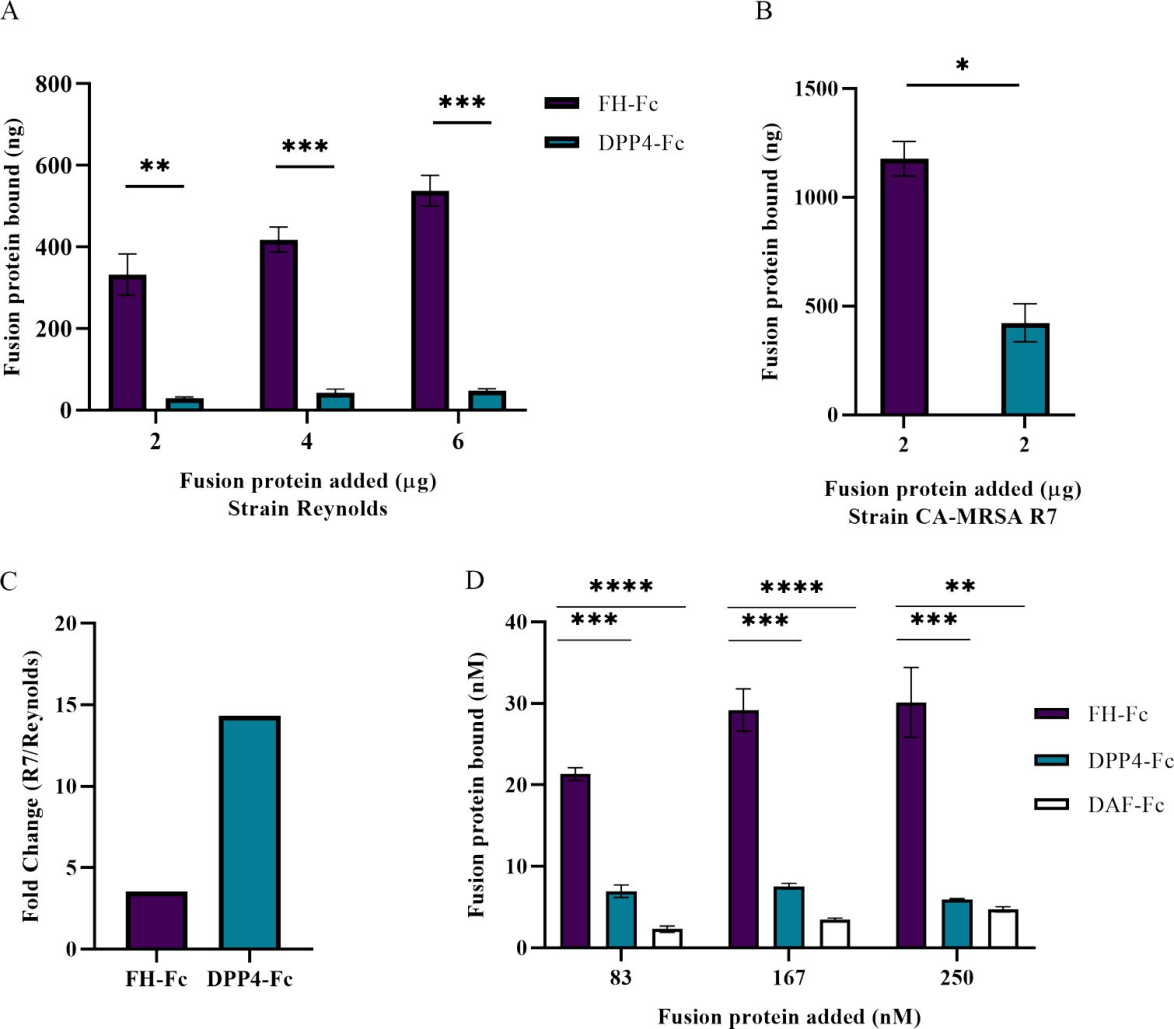
*S*. *aureus* binds significantly more FH-Fc than Fc-control protein. A, Fusion protein binding to lab strain Reynolds. B, Fusion proteins binding to CA-MRSA R7. C, Fold change of fusion protein binding to CA-MRSA R7 vs lab strain Reynolds (fusion protein added at 2μg). D, Fusion protein binding to CA-MRSA R7, nM comparison. Data represent the mean of at least three independent experiments ± SEM. * *p* < 0.05, ** *p* ≤ 0.004, *** *p* ≤ 0.0003.

The interaction between the fusion protein and *S*. *aureus* is dependent upon the structural properties of both interacting units, i.e., the *S*. *aureus* membrane protein and the fusion-protein regions (Fig 1). Thus, we investigated the ability of *S*. *aureus* to bind the candidate fusion protein (FH-Fc) compared to a variety of Fc- and FH-containing control proteins (see Table 1). *S*. *aureus* lab strain Reynolds bound significantly more FH-Fc compared to the control protein DPP4-Fc, as shown in Fig 2A. A similar binding trend was seen when testing CA-MRSA strain R7 (Fig 2B); however, R7 bound 3.5 fold more FH-Fc and 14.3 fold more DPP4-Fc than strain Reynolds (Fig 2C).

To examine the extent to which CA-MRSA R7 could bind FH-Fc and Fc-control proteins, we tested a range of fusion protein concentrations. As shown in Fig 2D, both DPP4-Fc and DAF-Fc were bound significantly less by *S*. *aureus* than FH-Fc for each concentration tested, demonstrating their utility as Fc-control proteins. To determine the effect of Protein A on fusion protein binding via the Fc region, we compared the binding ability of lab strain Newman and its *spA*-negative counterpart using FH-Fc, V1, and V2, which have identical FH regions but varied Fc domains. As shown in Table 2, fusion protein binding to *spA*-negative Newman (Protein-A deficient) was 8.2–13.4% less than for Newman Wildtype (WT), indicating that binding to the *S*. *aureus* surface via the Fc region, regardless of Fc-type tested, is minimal. Taken together, *S*. *aureus* bound fusion proteins containing FH (CCP18-20) regions significantly more than those without, highlighting FH (CCP18-20) as the dominant interacting domain with *S*. *aureus*.

**Table 2:** Fusion protein binding to *S*. *aureus* strain Newman (WT) compared to *S*. *aureus* Newman Protein A deficient (*spA* negative).

	*spA* negative/WT (% total binding)	*spA* negative/WT (% less binding)
FH-Fc (IgG1 Fc)	86.6% ± 6.1	13.4%
V1 (IgG1 Fc, D270A/K322A)	88.0% ± 1.9	12.0%
V2 (IgG3 Fc)	91.8 ± 12.8	8.2%

### FH-Fc binds to neutrophils

**Fig 3:**
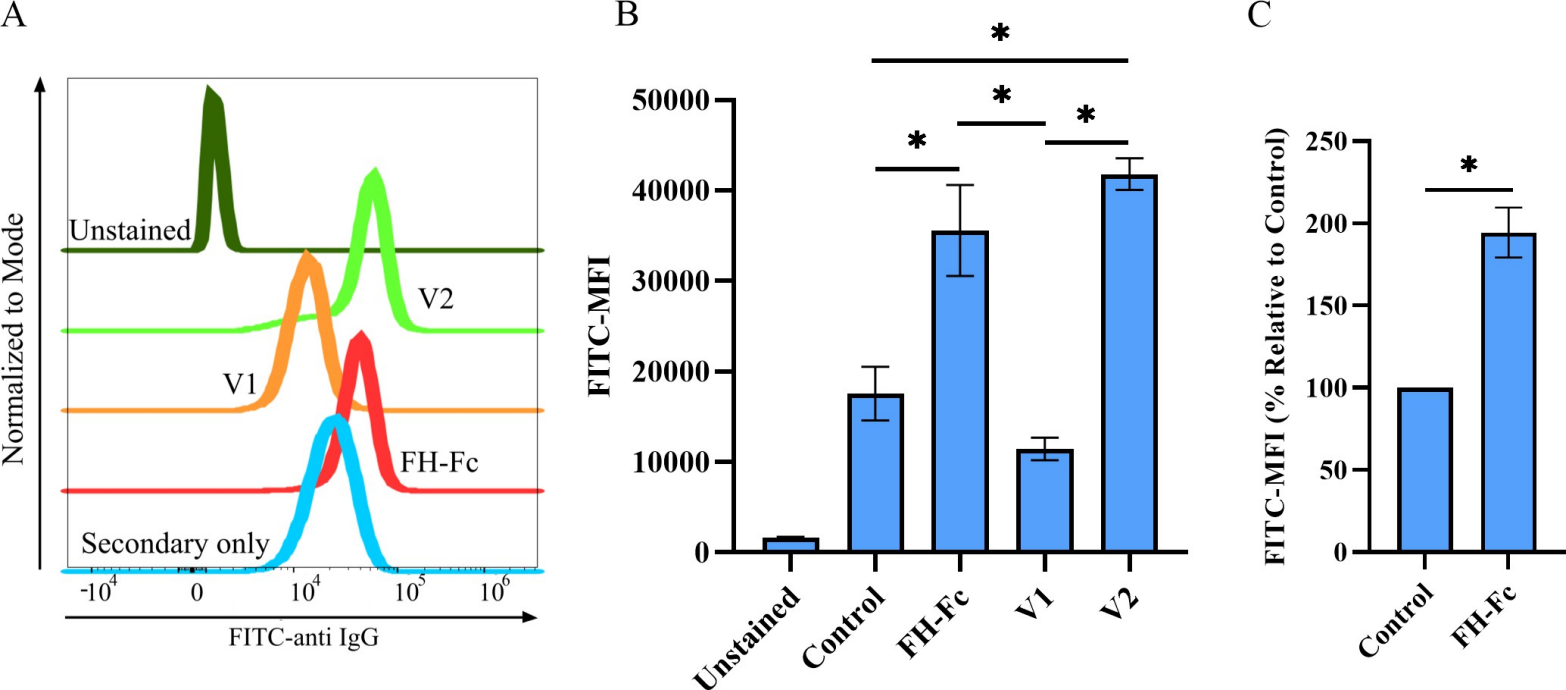
Fc region of fusion protein affects binding by phagocytes. Human peripheral blood leukocytes were incubated with 500 ng of FH-Fc, V1 or V2 for 15 minutes, stained for anti-CD15, neutrophils were gated as FSC^high^SSC^high^ CD15^+^ cells and analyzed for fusion-protein binding with goat anti-FH IgG followed by secondary donkey anti-goat IgG-FITC antibody by flow cytometry. A, Representative histogram is shown: unstained (olive green), V2 (bright green), V1 (orange), FH-Fc (red) and background control (blue, no fusion protein, secondary probe only). B, Mean fluorescence intensity (MFI) of anti IgG-FITC from three donors. C, Binding of FH-Fc relative to background control; n = 4 donors, 2 independent experiments. Data represent the mean ± SEM; controls were donor specific (* *p* < 0.05).

Binding by the FH region to the *S*. *aureus* surface presents an unbound Fc that is expected to interact with phagocytes. To confirm this interaction, we incubated FH-Fc, V1 or V2 with human peripheral blood leukocytes obtained from multiple healthy donors and assessed binding of fusion proteins to neutrophils by flow cytometry. Peripheral blood leukocytes from each individual donor were incubated with secondary probe only (no fusion protein or primary probe) and used as controls for background staining. As shown in a representative histogram from one donor, we detected a significant binding of FH-Fc and V2 but not V1 fusion protein to neutrophils (Fig 3A). The calculated average of detected mean fluorescence intensity (MFI) of anti-IgG-FITC antibody demonstrates that FH-Fc and V2 efficiently bind to human neutrophils from several donors (Fig 3B, and 3C).

### Normal human serum inhibits binding of FH-Fc to S. aureus R7

**Fig 4:**
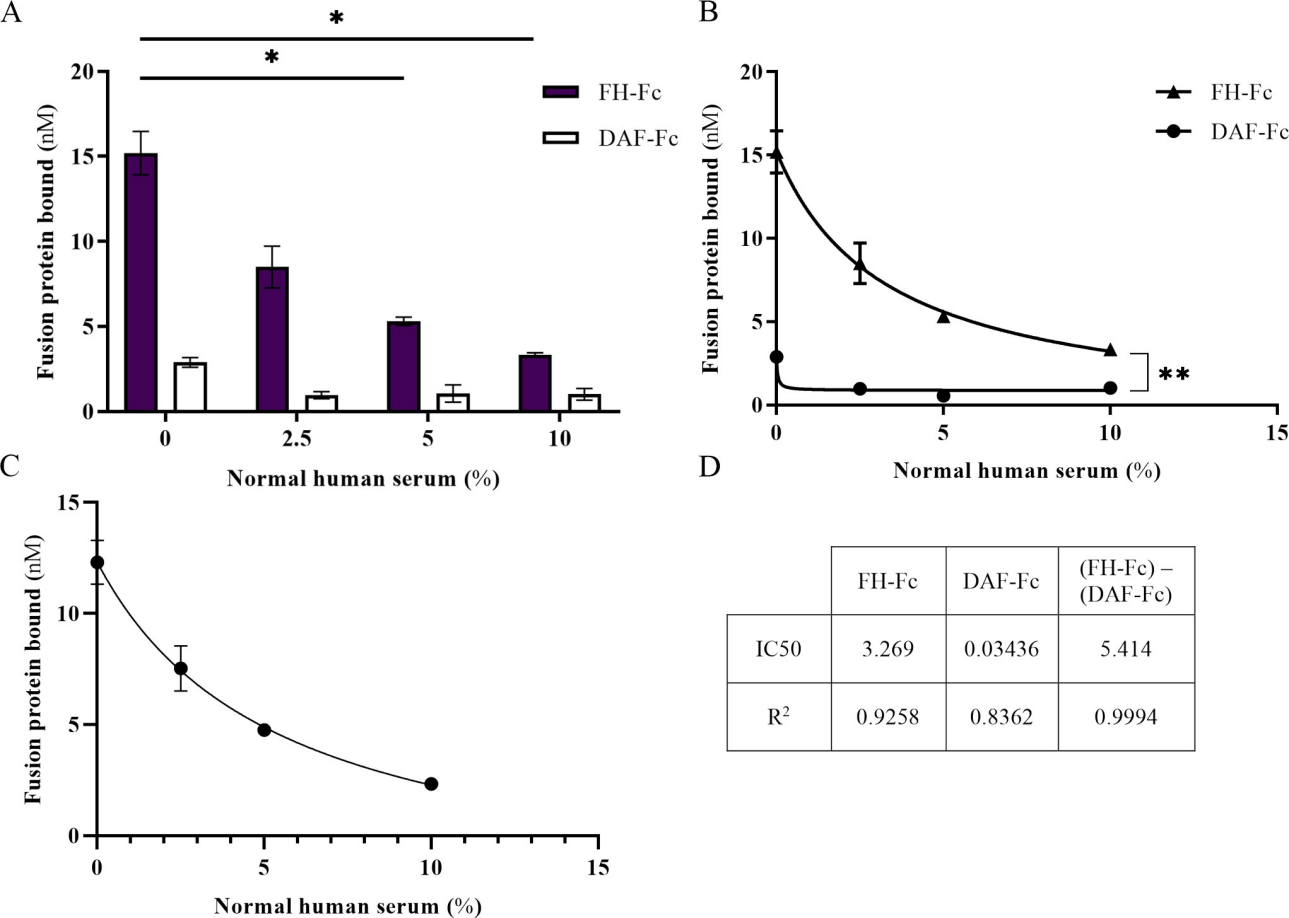
FH-Fc competes with serum for binding to CA-MRSA R7. A, Fluorescently labeled FH-Fc and DAF-Fc (62.5 nM) bound by R7 in the presence of increasing serum amounts, measured by fluorescence detection. B, Dose response curves of fluorescently labeled FH-Fc and DAF-Fc (62.5 nM) bound by R7 in the presence of serum. C, Dose-response curve of FH-Fc binding via FH-region, calculated by subtracting bound DAF-Fc from bound FH-Fc. D, IC50 and R^2^ of dose-response curves (B and C). Data represent a minimum of three independent experiments ± SEM, * *p* < 0.05, ** *p* ≤ 0.004.

Next, we sought to evaluate the extent to which normal human serum (NHS), a blood component that contains both antibody (Fc-containing proteins) and serum FH, affects FH-Fc binding to CA-MRSA R7. To directly assess fusion protein binding to *S*. *aureus*, we used click chemistry to label the N-linked glycans of both FH-Fc and a control protein with a small fluorescent dye (720 Da) to minimize label-induced binding hindrances. DAF-Fc was selected as the control for the fluorescent studies due to its glycosylation-site equivalence to that of FH-Fc. CA-MRSA R7 bacteria were incubated with a constant amount of labeled fusion protein (FH-Fc or control) in various amounts of NHS. As concentrations of NHS increased, a dose-dependent reduction in the binding of FH-Fc to CA-MRSA R7 was evident, with only minimal effect on DAF-Fc binding (Fig 4A and 4C). Dose-response curve data revealed that FH-Fc binding to CA-MRSA R7 was inhibited by 50% using 62.5 nM of FH-Fc and 3.269% of NHS (Fig 3D).

### FH-Fc inhibits binding of serum FH by S. aureus R7

**Fig 5:**
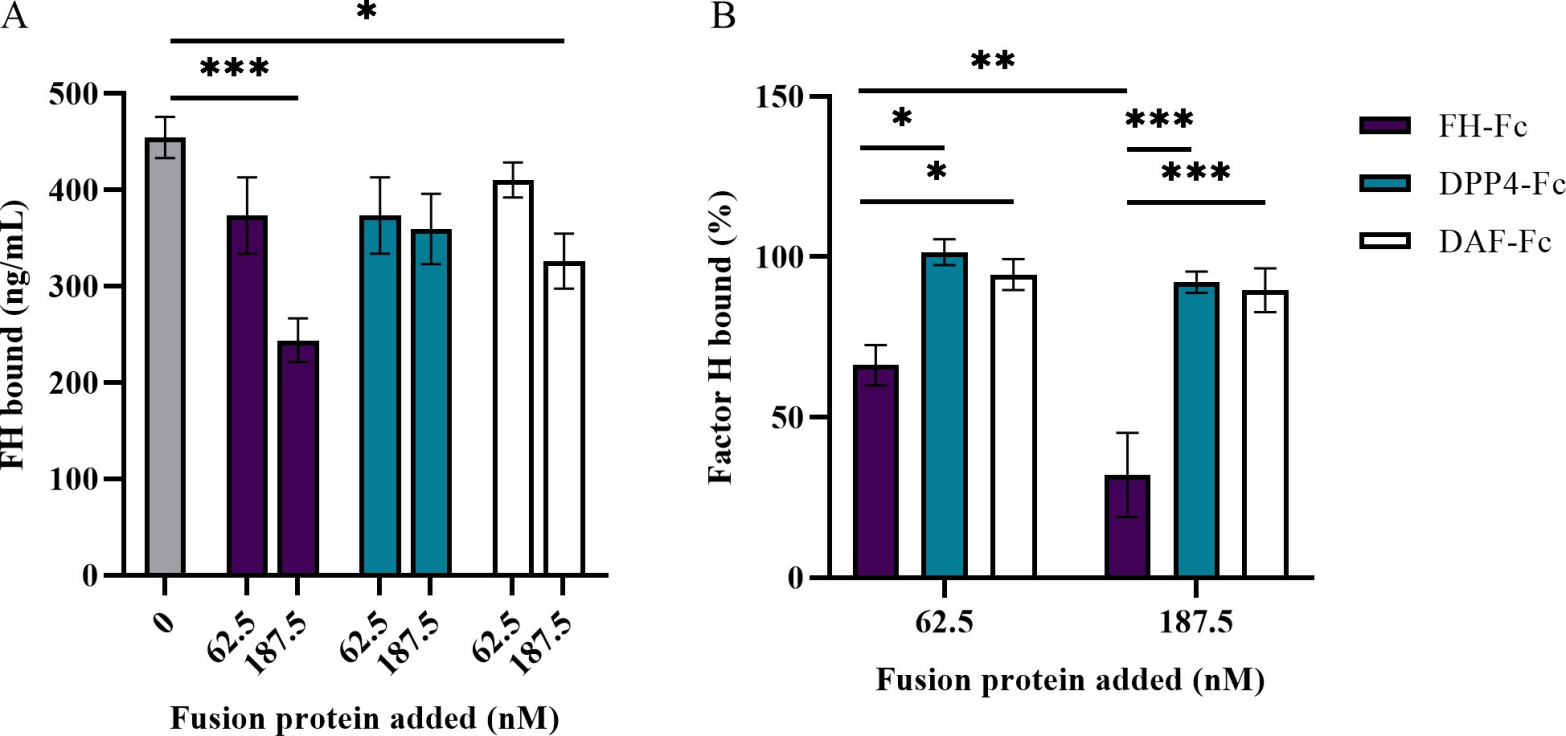
Serum FH competes with fusion proteins for binding to *S*. *aureus*. CA-MRSA R7 was incubated in 2.5% NHS and fusion protein. Bound protein was extracted and evaluated. A, ELISA quantitation of bound serum FH, probing with mAb anti CCP1-4 (not found on FH-Fc). B, Optical densitometry measurement of FH bands visualized on anti-FH Western blot of bound serum FH, calculated as a percentage of serum FH bound compared to no fusion protein control. Data represent the mean of at least three independent experiments ± SEM. * *p* < 0.05, ** *p* ≤ 0.004, *** *p* ≤ 0.0003.

To confirm that serum FH competes with FH-Fc for *S*. *aureus* binding, CA-MRSA R7 was incubated with increasing amounts of FH-Fc or control protein and a constant amount of NHS. In 2.5% NHS, 18 μg/mL (187.5 nM) of FH-Fc significantly reduced serum FH bound to CA-MRSA R7, compared to no fusion protein or DPP4-Fc; DAF-Fc treatment also reduced serum FH binding but to a lesser degree than FH-Fc (Fig 5A). The reduction in serum FH binding when treated with FH-Fc was confirmed by Western-blot analysis, as measured by optical densitometry of FH bands, and calculated as a percentage of no-treatment control. FH-Fc treatment at both 62.5 nM and 187.5 nM in 2.5% NHS resulted in a significant reduction in bound serum FH, whereas neither control protein exerted a significant effect on FH binding (Fig 5B). These results suggest that FH-Fc competes with serum FH for binding by SdrE, and that FH CCP18-20 is sufficient to establish a binding affinity for SdrE that is competitive with full length FH.

### FH-Fc and V1 increase C3-fragment deposition of S. aureus R7

**Fig 6:**
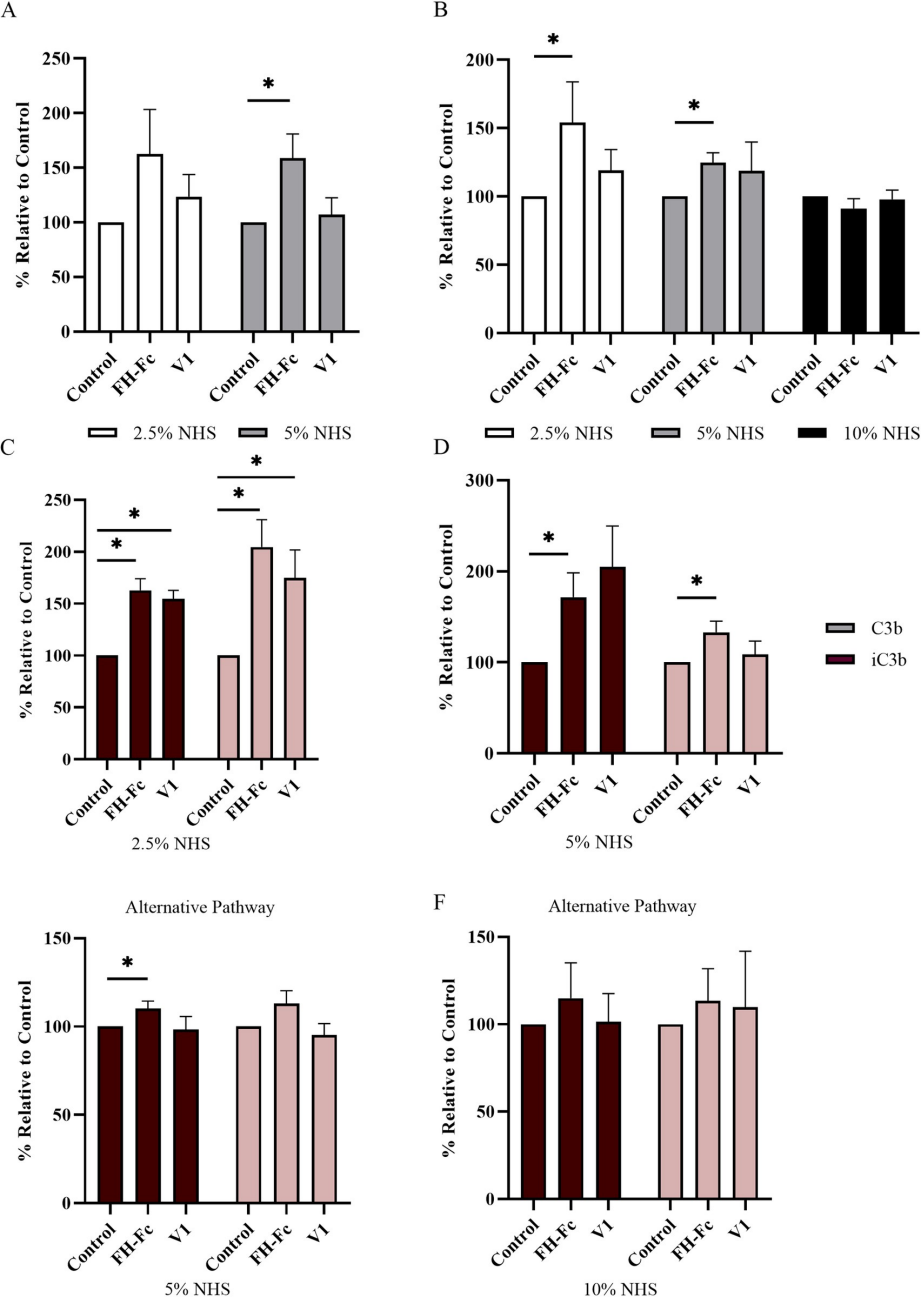
FH-Fc and V1 increase C3-fragment deposition of CA-MRSA R7. Bacteria (5 × 10^7^ CFU) were incubated with or without 4.5μg fusion protein in various amounts of NHS for 30 minutes. Deposited C3-fragments were extracted and analyzed by ELISA (total C3) and Western Blot (C3b and iC3b). Total C3-fragment deposition for all complement pathways (A) and alternative pathway only (B). C-F, Western blot semi-quantification of samples tested in (A) and (B) presented as a percent relative to no-treatment control. Data represent mean ± SEM of at least 3 independent experiments; * *p* < 0.05.

In addition to competing with serum FH for binding by *S*. *aureus*, FH-Fc binding via the FH(18–20) region exposes an available Fc (Fig 1D) permitting a potential interaction with C1q, the initiating protein of the classical pathway. Thus, to test whether fusion protein binding affects complement activation, we measured the level of C3-fragment deposition on the surface of CA-MRSA R7. To permit the direct assessment of the exposed Fc region, V1 (with abrogated ability to activate C1q) was used as a control [29, 35]. As shown in Fig 6A, FH-Fc and V1 treatment caused an increase in total C3-fragment deposition in 2.5% NHS, which was significant in 5% NHS when treated with FH-Fc compared to control (*p* = 0.04). Under conditions that permitted activation of the alternative pathway only, total C3-fragment deposition was significantly increased in both 2.5% and 5% NHS when treated with FH-Fc; however, no significant effect was detected in 10% NHS (Fig 6B). Extracts were further evaluated for C3b and iC3b levels via Western blot revealing a significant increase for both C3b and iC3b in 2.5% NHS treated with either FH-Fc or V1 compared to control (Fig 6C, *p* = 0.034). This effect was maintained for FH-Fc treatment in 5% NHS (Fig 6D, *p* = 0.037). When examining C3b or iC3b deposition via the alternative pathway, C3b deposition was significantly increased in 5% NHS treated with FH-Fc (Fig 6E, *p* = 0.034); C3-fragment extracts generated by the alternative pathway in 2.5% NHS were insufficient to evaluate by western blotting; no statistical difference was detected for samples generated by the alternative pathway in 10% NHS (Fig 6F).

### FH-Fc increases C5a generation

**Fig 7:**
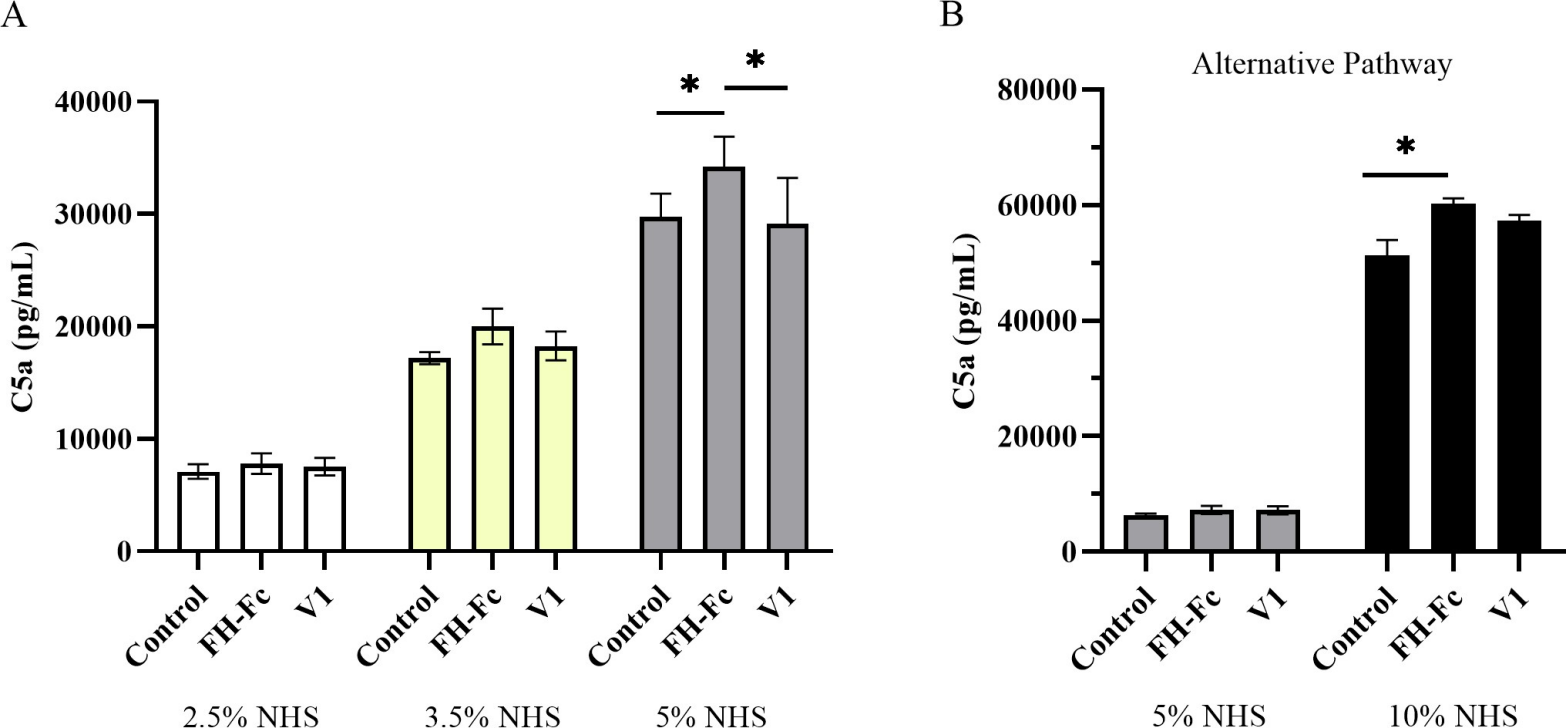
FH-Fc treatment increases C5a generation. CA-MRSA R7 (5 × 10^7^ CFU) were incubated with various amounts of NHS ± 4.5 μg fusion protein for 15 minutes. The supernatant was analyzed for the presence of C5a via ELISA. A, All pathways active. B, Alternative pathway only. Data represent the mean ± SEM of at least four independent experiments (* *p <* 0.03).

Although an increase in C3-fragment deposition indicates heightened complement activation, both C3b and iC3b deposition on *S*. *aureus* were significantly increased. As iC3b cannot participate in complement amplification, we investigated how FH-Fc treatment affects the downstream generation of the anaphylatoxin C5a using various concentrations of NHS and a constant amount of fusion protein and CA-MRSA R7. FH-Fc treatment in 2.5% NHS and 3.5% NHS caused no significant effect on C5a generation; however, in 5% NHS, FH-Fc treatment significantly increased C5a generation compared to V1 treatment or no fusion protein control (Fig 7A, *p* < 0.03). In conditions that permitted activation of the alternative pathway only, C5a generation was significantly increased in 10% NHS when treated with FH-Fc (Fig 7B, *p* = 0.0157).

### FH-Fc significantly reduces survival of S. aureus

**Fig 8:**
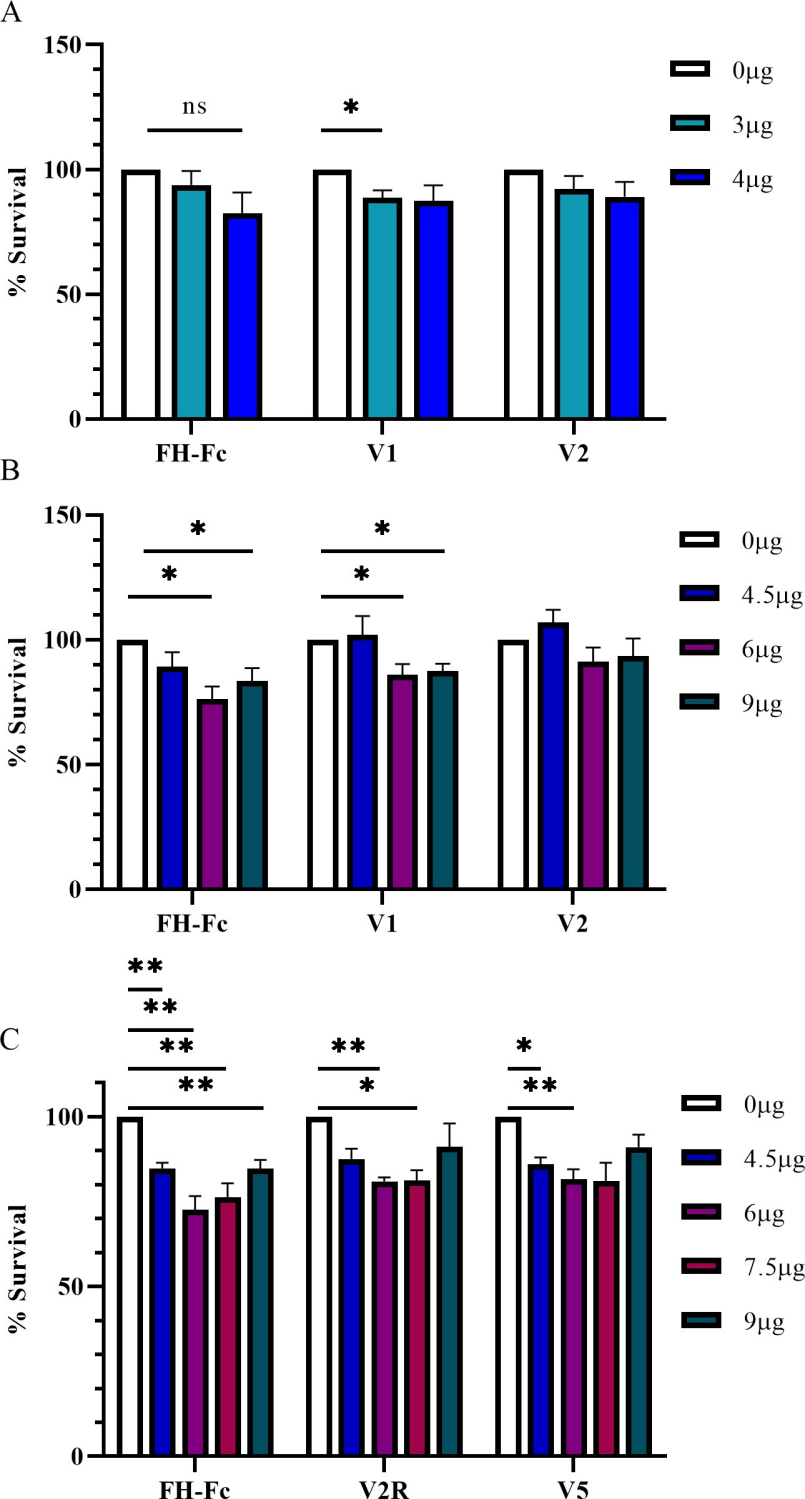
Fusion protein treatment reduces survival of CA-MRSA R7. CA-MRSA R7 were incubated with NHS ± fusion protein for 15 minutes then challenged with polymorphonuclear cells at a ratio of 1 PMN per 10 bacteria. After rotating at 37°C for 75 minutes, samples were serially diluted and plated. A, In 2.5% NHS. B, In 5% NHS. C, In 10% NHS. Data represent the mean ± SEM of at least four independent experiments (* *p* < 0.05, ** *p* ≤ 0.004).

An increase in C3-fragment deposition and C5a generation signify an increase in complement activation, and consequently a reduction in complement evasion. Thus, we sought to determine the effect of fusion proteins on the survival of *S*. *aureus* when challenged with PMNs. CA-MRSA R7 were incubated with various concentrations of NHS and fusion protein, and challenged with PMNs at a ratio of 1 PMN per 10 bacteria. In 2.5% NHS, FH-Fc showed the greatest effect on survival (17.5% reduction); however, this result was not significant (Fig 8A). In 5% NHS, both FH-Fc and V1 reduced survival of CA-MRSA R7 compared to control (Fig 8B). Although the reduction was slight (14–21% reduction in survival), these results were statistically significant (*p* ≤ 0.01).

We next compared the effect of FH-Fc and two new variants, V2R and V5 on *S*. *aureus* survival in 10% NHS and challenged with PMNs. V2R is comprised of the same components as V2; however, the orientation of FH and Fc regions is reversed. V5 is the same as V2R except for an EFT modification in the Fc region that has been shown to improve binding to C1q. Again, FH-Fc showed the greatest reduction in survival (6 μg FH-Fc, 27.4% reduction in survival) compared to control (Fig 8C).

To further examine the anti-staphylococcal effect of FH-Fc treatment, we tested three additional CA-MRSA USA300 clinical isolates using FH-Fc, V2R and V5. As shown in Table 3, all three fusion proteins were effective in significantly reducing survival for 75% (3/4) of the CA-MRSA isolates tested in 10% NHS; one strain appeared to resist treatment. To investigate whether there were any differences in binding of FH-Fc across these isolates, we performed a binding assay, as before. As shown in Table 4, the strain that appeared resistant to FH-Fc treatment, CA-MRSA 17, was shown to have a reduced ability to bind FH-Fc compared to the other isolates tested; however, this was not statistically significant.

**Table 3:** CA-MRSA percent survival in 10% NHS with PMNs; fusion-protein treatment relative to no-treatment control.

	CA-MRSA R7	CA-MRSA 17	CA-MRSA 22	CA-MRSA 26
**FH-Fc: 6 μg**	72.6% ± 3.1*	104.0% ± 13	74.0% ± 10.1	82.1% ± 5.4*
**7.5 μg**	76.2% ± 3.8*	103.4% ± 4.3	78.4% ± 4.3*	73.1% ± 6.1*
**V2R: 6 μg**	80.9% ± 2.4**	102.4% ± 11	89.1% ± 11.8	77.1% ± 10.7
**7.5 μg**	81.2% ± 3.6	99.4% ± 7.3	80.8% ± 5.6*	87.5% ± 7.4
**V5: 6 μg**	81.5% ± 2.4*	105.0% ± 6	84.1% ± 1.9*	87.0% ± 4.4*
**7.5 μg**	81.0% ± 3.2	114.9% ± 11	73.4% ± 7*	91.1% ± 4

Data represent mean ± SEM of at least three independent experiments

* *p <* 0.05

** *p* ≤ 0.004.

**Table 4:** FH-Fc binding to various CA-MRSA isolates.

	FH-Fc bound (ng)	Binding compared to CA-MRSA R7
CA-MRSA R7	448.7 ± 31.5	100%
CA-MRSA 17	357.4 ± 33.9	79.6% ns
CA-MRSA 22	444.5 ± 42.7	99.1% ns
CA-MRSA 26	441.3 ± 31.6	98.3% ns

Data represent mean ± SEM of three independent experiments, ns (not significant).

## Discussion

As the leading cause of both community and healthcare-associated infections, and with antibiotic resistance on the rise, novel anti-staphylococcal treatments are in critical need [2]. As such, we examined the extent to which FH-Fc fusion proteins, containing two potential binding regions to block *S*. *aureus* virulence factors, affected complement-mediated opsonophagocytosis and killing of CA-MRSA clinical isolates. Of note, CA-MRSA R7, the principal isolate tested, has previously been shown to bind serum FH [6].

The principal fusion protein, FH-Fc, was bound significantly more by lab strain Reynolds and CA-MRSA R7 than Fc-control proteins, indicating FH(18–20) as the dominant binding domain. Interestingly, the scale at which lab strain Reynolds bound FH-Fc or the control protein DPP4-Fc was considerably less than binding by CA-MRSA R7, which may be attributed to expression differences of SdrE and Protein A/Sbi associated with the lack of evolutionary pressures imposed on lab strains. To further examine the dominant binding confirmation of FH-Fc by *S*. *aureus*, we tested the binding of FH-Fc, V1, and V2, possessing identical FH regions but dissimilar Fcs, using lab strain Newman and Protein A-deficient Newman (*spA* negative). Although Newman bound more fusion protein than *spA*-negative Newman, the difference was not statistically significant, substantiating FH(18–20) as the dominant binding domain.

In serum, FH-Fc competed for *S*. *aureus* binding such that the amount of NHS required to inhibit 50% of fusion protein binding was almost 100-fold more for FH-Fc compared to the Fc-control protein, DAF-Fc. Likewise, increasing amounts of FH-Fc competitively inhibited serum FH binding by *S*. *aureus*. Compared to no fusion protein, 187.5 nM of FH-Fc reduced serum FH binding by 46.3%, whereas 187.5 nM of DAF-Fc reduced serum FH binding by 28.2%, as measured by ELISA. The inhibitory effect of FH-Fc on serum FH recruitment by *S*. *aureus* was confirmed by Western blotting, though with an amplified effect, which may be attributed to the semi-quantitative nature of this technique. Due to a far greater proportion of IgG in NHS compared to FH (7–16 mg/mL IgG versus 116–562 μg/mL FH) [36, 37], it is likely that competition for FH-Fc binding by *S*. *aureus* would be inherently greater for the Fc region. Thus, any application of FH-Fc fusion proteins in a serum-encompassing environment will require a higher degree of affinity for the FH region over the Fc component, when there is potential for Fc-binding.

The predominance of SdrE:FH-Fc binding supports our expectation of increased complement activation as measured by an increase in C3-fragment deposition. Although V1 contains an altered Fc region rendering it unable to interact with C1q, the effect of FH-Fc and V1 were similar with respect to opsonizing levels of C3b and iC3b on CA-MRSA R7, when all complement pathways were active in 2.5% NHS; however, in 5% NHS, FH-Fc outperformed V1 in its ability to increase C3-fragment opsonization of CA-MRSA R7, indicating that the Fc region plays a role in complement activation. As C1q has a low affinity for singular IgG and requires the binding of multiple IgG molecules to activate [38], the surface clustering of SdrE, and subsequent recruitment of FH-Fc by SdrE may be insufficient to provide the multimeric scaffold of available Fc regions capable of directly activating the classical pathway of complement in low amounts of serum. To circumvent the effect of the Fc region on complement activation, we repeated these assays under conditions that permitted activation of the alternative pathway only; as it typically requires higher concentrations of serum to activate, 10% NHS was included as a condition. FH-Fc treatment enhanced C3-fragment opsonization compared to no treatment in both 2.5% and 5% NHS, with an increase in C3b opsonization observed in 5% NHS. In contrast to conditions permitting all pathways, no statistical significance was detected between FH-Fc and V1 treatments, supporting that the Fc region augments complement activation when all pathways are permitted. Due to the rampant nature of complement, the power of the alternative pathway in 10% NHS was likely too strong for the applied fusion protein to yield any detectable effect.

Opsonizing C3b can contribute to further amplification of the complement cascade through the generation of additional C3-convertases (C3bBb, of the alternative pathway) or C5 convertases (C4bC2aC3b or C3bBbC3b, of the classical/lectin and alternative pathways, respectively), whereas iC3b, an inactive form of C3b, cannot [39]. Thus, we examined the extent to which FH-Fc and V1 affected the generation of the anaphylatoxin C5a. In 5% NHS, treatment with FH-Fc caused a significant increase in C5a generation compared to either V1 or no treatment groups when all pathways were active. Under conditions supporting activation of the alternative pathway only, FH-Fc treatment in 10% NHS significantly increased C5a generation compared to untreated control, with no statistical significance between FH-Fc and V1. These data support that FH-Fc treatment augments complement activation.

Although iC3b cannot contribute to complement amplification, iC3b is a major opsonin, similar to C3b. As such, both C3b and iC3b are recognized by complement receptors on phagocytes (reviewed in [40]). Taken together, an increase in C3-fragment opsonization and C5a generation support the probability that FH-Fc treatment would also increase complement-mediated opsonophagocytosis of *S*. *aureus* leading to greater bacterial killing. Indeed, when incubated in 5% NHS and human PMNs, a significant reduction in *S*. *aureus* survival was observed for groups treated with either FH-Fc or V1 (6 μg and 9 μg). V1, with a mutated Fc previously shown to have diminished capacity to bind FcγRs [41], was unable to sufficiently bind neutrophils; thus, the anti-staphylococcal activity of V1 is likely attributed to an enhancement of C3-fragment opsonization through serum FH displacement. Surprisingly, treatment with V2, which possesses an IgG3 Fc with an expected higher affinity for C1q and demonstrated interaction with neutrophils, resulted in no significant effect.

To expand our testing of FH-Fc fusion proteins on the survival of *S*. *aureus*, we next introduced two additional constructs, one similar to V2 but with a reversed orientation (V2R), the second similar to V2R but with a modified Fc designed to improve the binding affinity for C1q. In 10% NHS, all three fusion proteins (FH-Fc, V2R and V5) were able to significantly decrease the survival of CA-MRSA R7 using 6 μg of fusion protein. However, FH-Fc was successful in significantly reducing CA-MRSA R7 survival for all 4 doses tested (4.5, 6, 7.5, 9 μg), with 6 μg having the greatest anti-staphylococcal effect (27.4% reduction in survival). Curiously, as the dose increased beyond 6 μg, treatment became less efficacious. As *S*. *aureus* can sense and adapt to changes in its environment, fusion-protein levels that surpassed this seemingly efficacious dose may have caused a differential shift in staphylococcal virulence factor expression to promote bacterial survival.

To more comprehensively assess the effect of FH-Fc, we tested three additional CA-MRSA clinical isolates that, like CA-MRSA R7, were of sequence type 8, SCC*mec* type IVa, and Panton-Valentine leukocidin positive, all key characteristics of USA300, the predominant CA-MRSA type in the US. In 10% NHS, all three fusion proteins (FH-Fc, V2R and V5) were effective at significantly reducing the survival of 3 of the 4 isolates tested (including CA-MRSA R7). One isolate, CA-MRSA 17, appeared to be resistant to fusion-protein treatment, which may be attributed to its lower capacity to bind FH-Fc compared to the other isolates. Although all CA-MRSA isolates tested possess *sdrE*, the expression of SdrE, and indeed other virulence factors, likely varies across strains leading to the varying effect on survival. Of note, additional *S*. *aureus* surface proteins with N-terminal domains similar to SdrE may also bind FH. However, whether these regions bind CCP19-20, has not been determined [42].

*S*. *aureus* is well known for its ability to evade phagocytosis, including its anti-phagocytic capsule and secreted proteins such as SCIN and CHIPS (chemotaxis inhibitory protein of *Staphylococcus*) that block complement-dependent phagocytosis and neutrophil chemotaxis, respectively [43, 44]. Moreover, *S*. *aureus* can escape the caustic environment of the PMN through mechanisms such as bacterial surface charge modification to repel host defensins, the production of cytolytic toxins that directly kill PMNs (e.g., Panton-Valentine leukocidin), and proteins and enzymes that protect against reactive oxygen species (reviewed in [45]). In this study, all CA-MRSA strains tested were *pvl* positive, indicating their potential to lyse PMNs directly. Whilst a direct assessment of fusion-protein effect on PMN chemotaxis or PMN survival was not measured, the impact of fusion-protein treatment on *S*. *aureus* survival supports the notion that FH-Fc and its derivatives (V2R and V5) were successful in diminishing *S*. *aureus* immune-evasion mechanisms such that *S*. *aureus* became more susceptible to killing by PMNs.

In typical virulence-factor-targeting anti-staphylococcal strategies, the removal of an immune-evasive or infection-progressing protein is met with the immediate implementation of a replacement, due to staphylococcal multi-purposing of virulence factors and their inherent redundancy [46]. Strategies that target the *S*. *aureus* quorum-sensing system through the accessory gene regulator (*agr*), the mechanism by which a burgeoning infection switches from adhesion to invasion based on *S*. *aureus* cell density, have been ineffective due to the pathogen’s ability to adapt [47]. Similarly, vaccine development has been unsuccessful due to complications with targeting staphylococcal virulence factors, as *S*. *aureus* expresses different systems of immune evasion and invasion mechanisms depending on the infection environment. The host-targeted nature of staphylococcal virulence factors makes vaccines especially prone to immuno-pathological responses [48].

Accordingly, FH-Fc fusion proteins are designed to function as somewhat of a multi-tool, containing alternate regions to counteract the redundancy imposed by *S*. *aureus* with abrogated ability to bind to C3b-coated host surfaces. Our data support that binding by the FH region, which competes with serum FH, permits an interaction between the exposed Fc region and FcγRs, and encourages the inflammatory response through complement activation and phagocytosis. With the potential to block immune-evasive proteins, FH-Fc mitigates the protective effects of bound serum FH and, to a lesser degree IgG bound by Fc, rendering *S*. *aureus* more vulnerable to the host immune system.

As seen in previous fusion-protein studies, an immuno-mimetic design typically results in low toxicity whilst recruiting the natural assistance of the host immune system [49]; therefore, therapeutics of this nature are less likely to cause adverse reactions in humans. Due to widespread antibiotic resistance, there is a strong need for effective and safe options that do not contribute to the growing problem of multidrug resistance. Future studies will focus on improving the efficacy and greater applicability of fusion proteins as potential anti-staphylococcal agents.

## Supporting information

S1 Data (XLSX)

S1 Raw images (DOCX)
