# Supplementary material for: A Factor H-Fc fusion protein increases complement-mediated opsonophagocytosis and killing of community associated methicillin-resistant Staphylococcus aureus
Source: PLoS One. 2022 Mar 24;17(3):e0265774. doi: 10.1371/journal.pone.0265774 (PMC8946749; doi:10.1371/journal.pone.0265774)

Fig 2A Raw Data


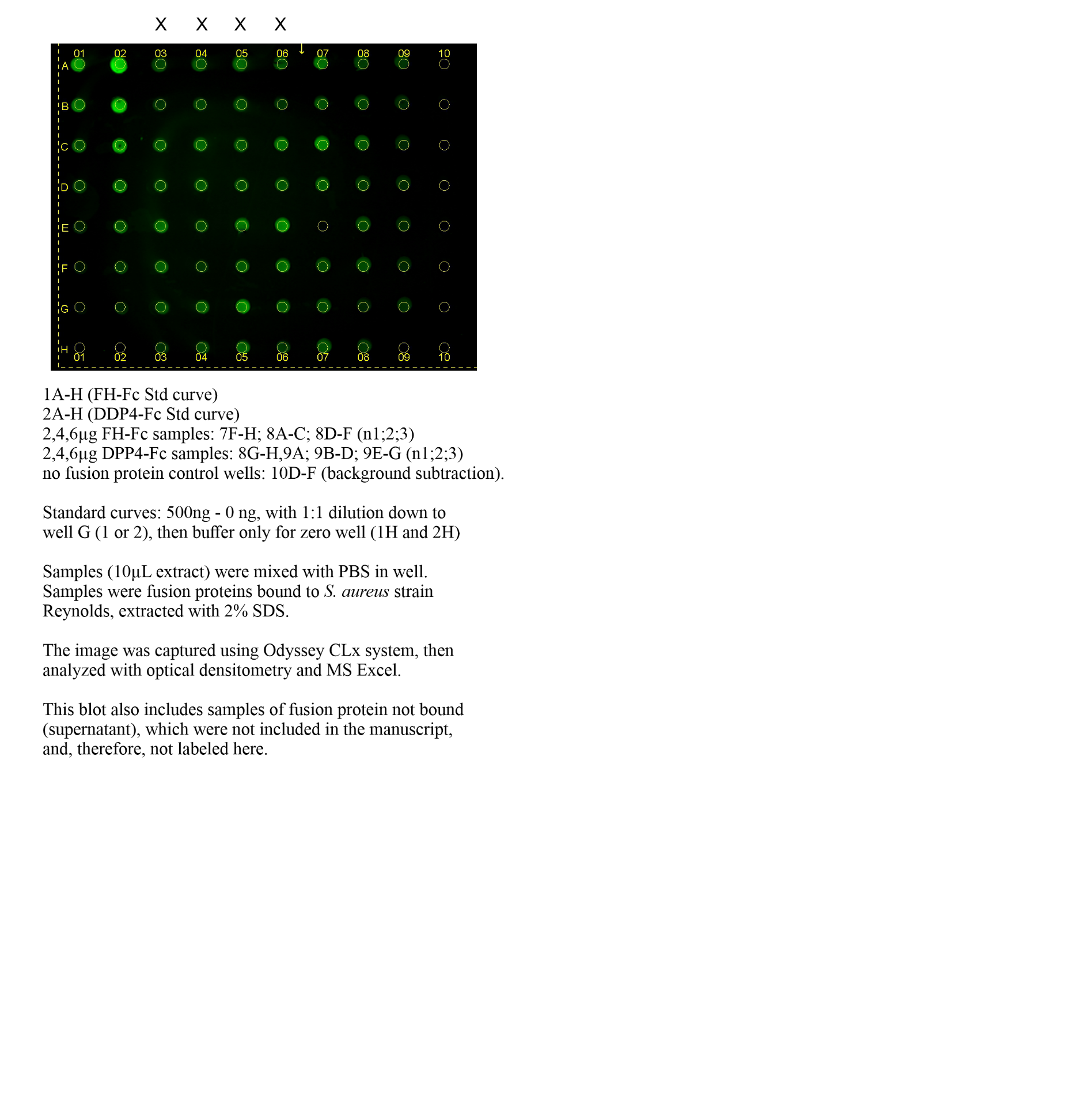


Fig 2B,D Raw Data


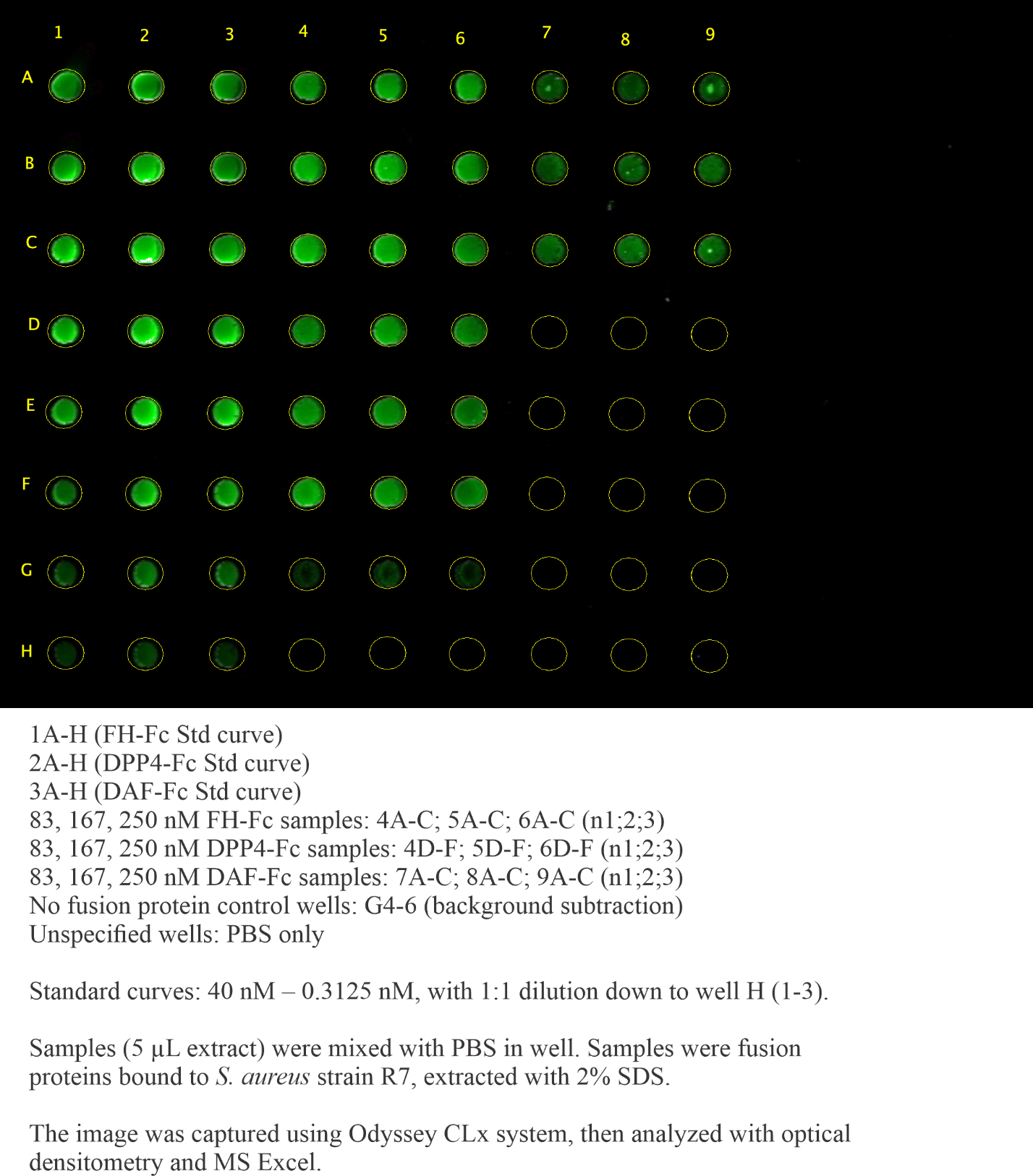


|  | D1 | D2 | D3 | D4 | AVG | SEM |  |  |
| --- | --- | --- | --- | --- | --- | --- | --- | --- |
| Unstained | | 1697 | 1404 | 1782 | 1627.667 | 114.4936 |  |  |
| Control | 53747 | 20607 | 11581 | 19610 | 26386.25 | 2857.033 |  |  |
| FH-Fc | 85753 | 36820 | 25767 | 41328 | 47417 | 4622.623 |  |  |
| V1 |  | 12520 | 9011 | 12374 | 11301.67 | 1146.109 |  |  |
| V2 |  | 40437 | 44459 | 37750 | 40882 | 1949.461 |  |  |

Figure 3, Raw Data (FACS, Mean Fluorescence Intensity – FITC, geometric mean)


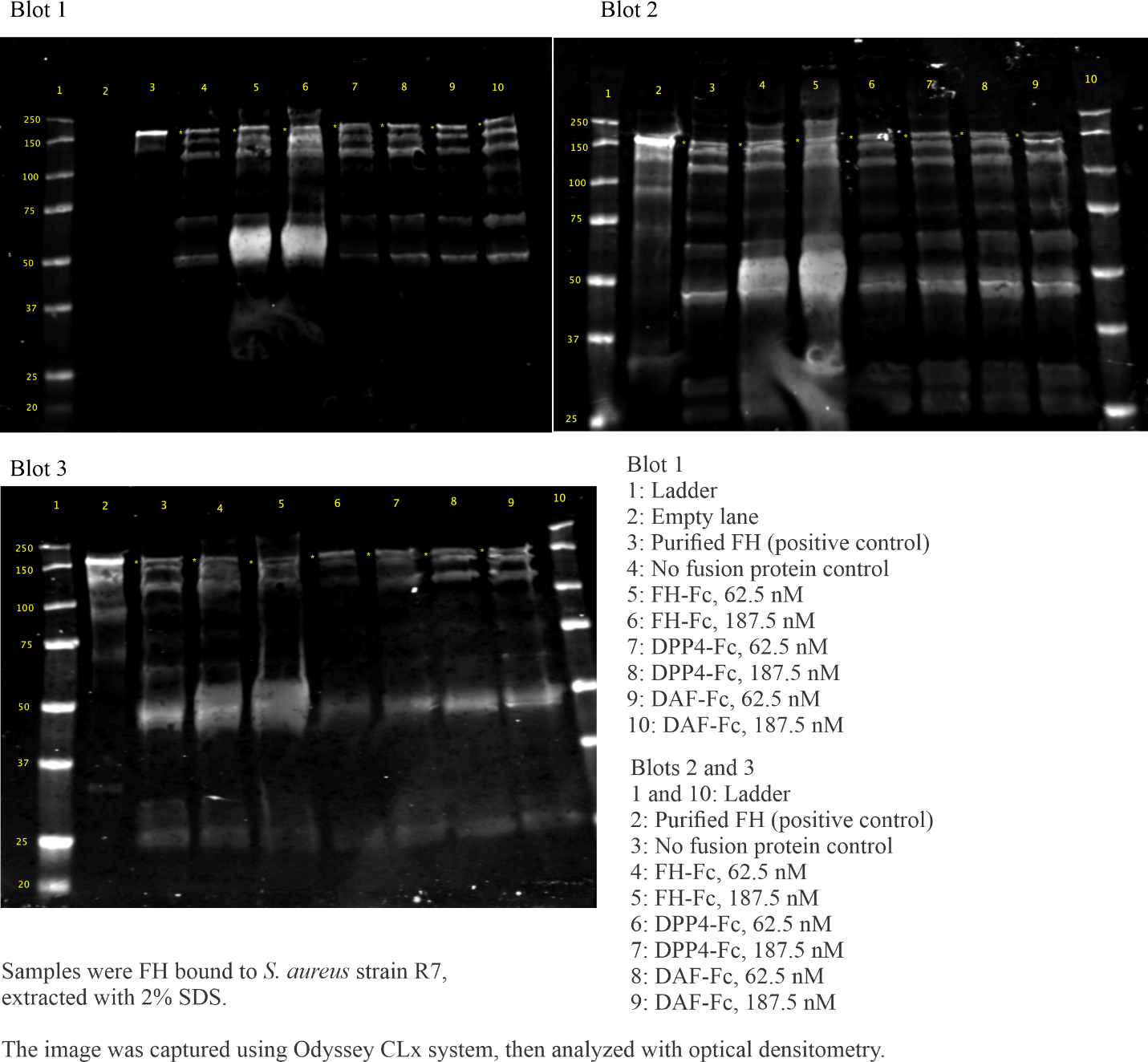
Figure 5B Raw Data


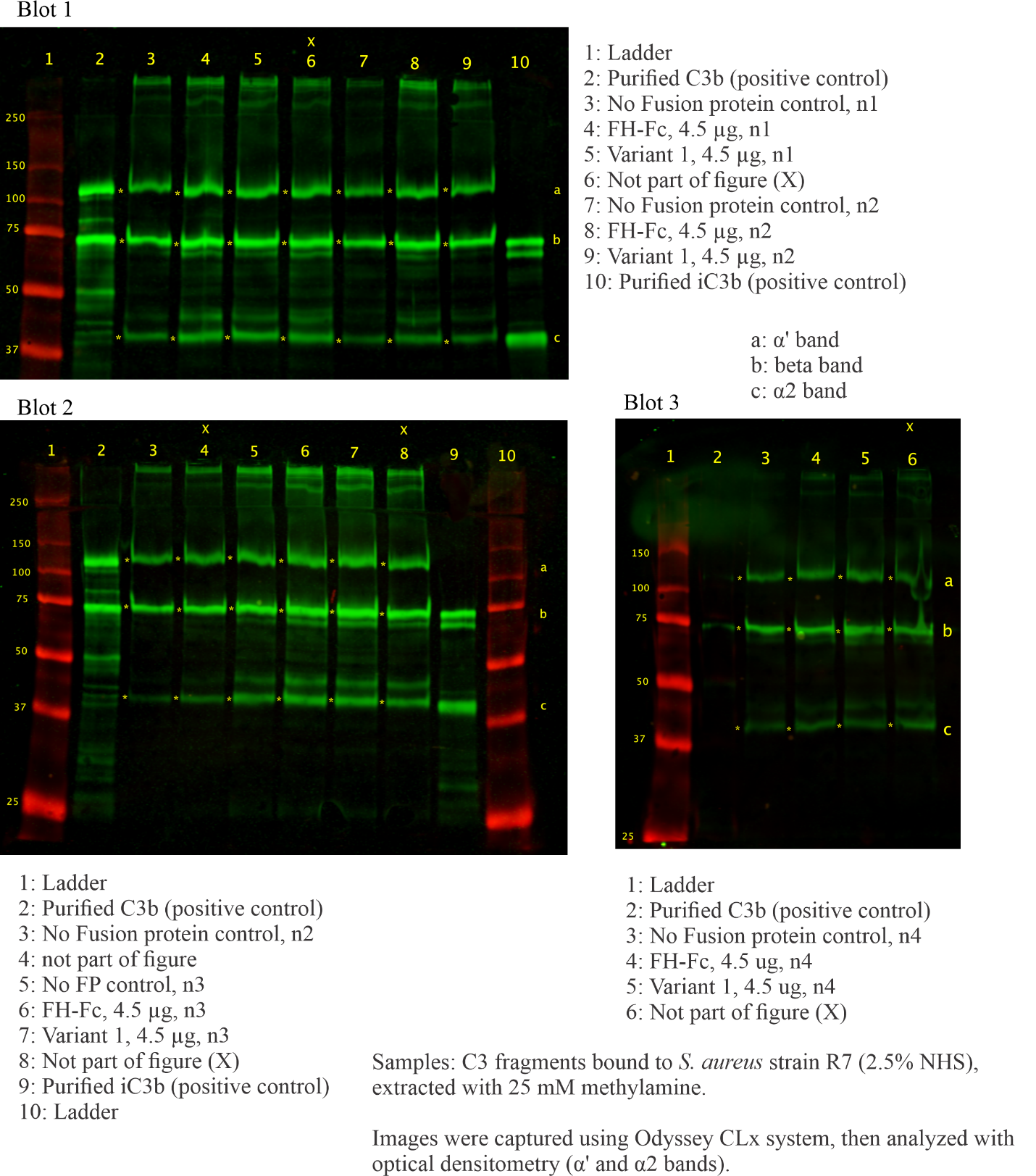
Figure 6C Raw Data

Figure 6D Raw Data


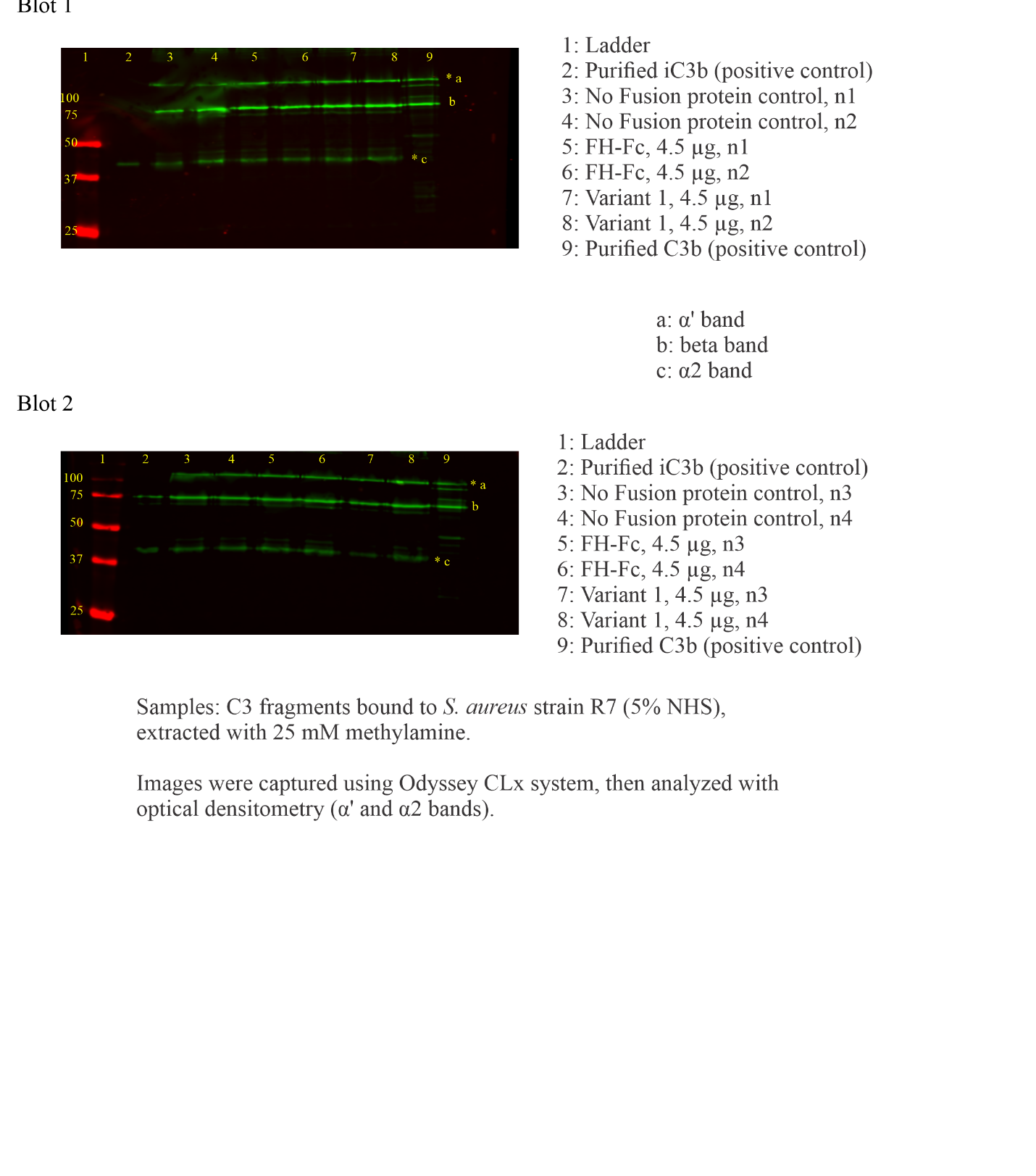


Figure 6E Raw Data


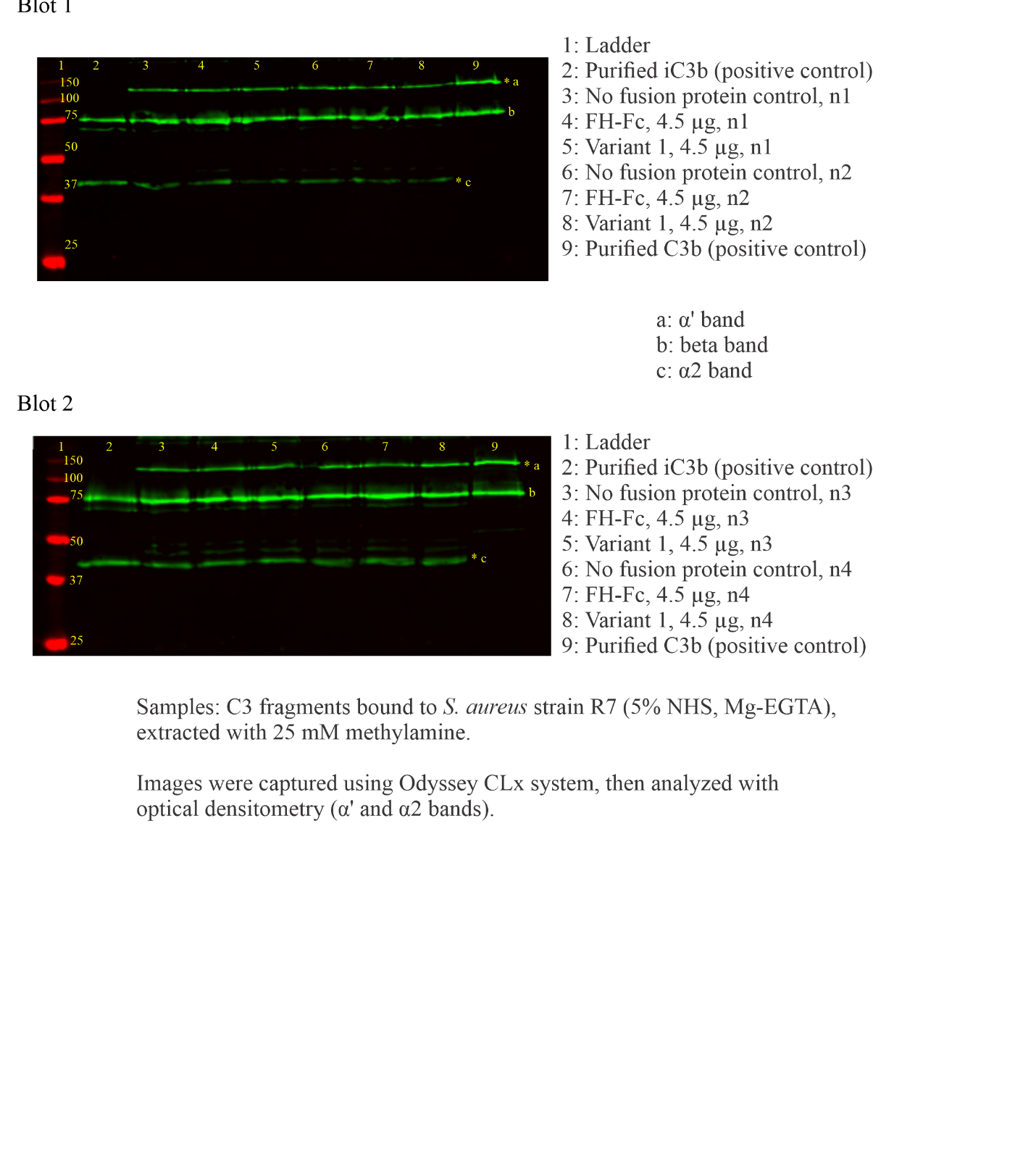


Figure 6F Raw Data


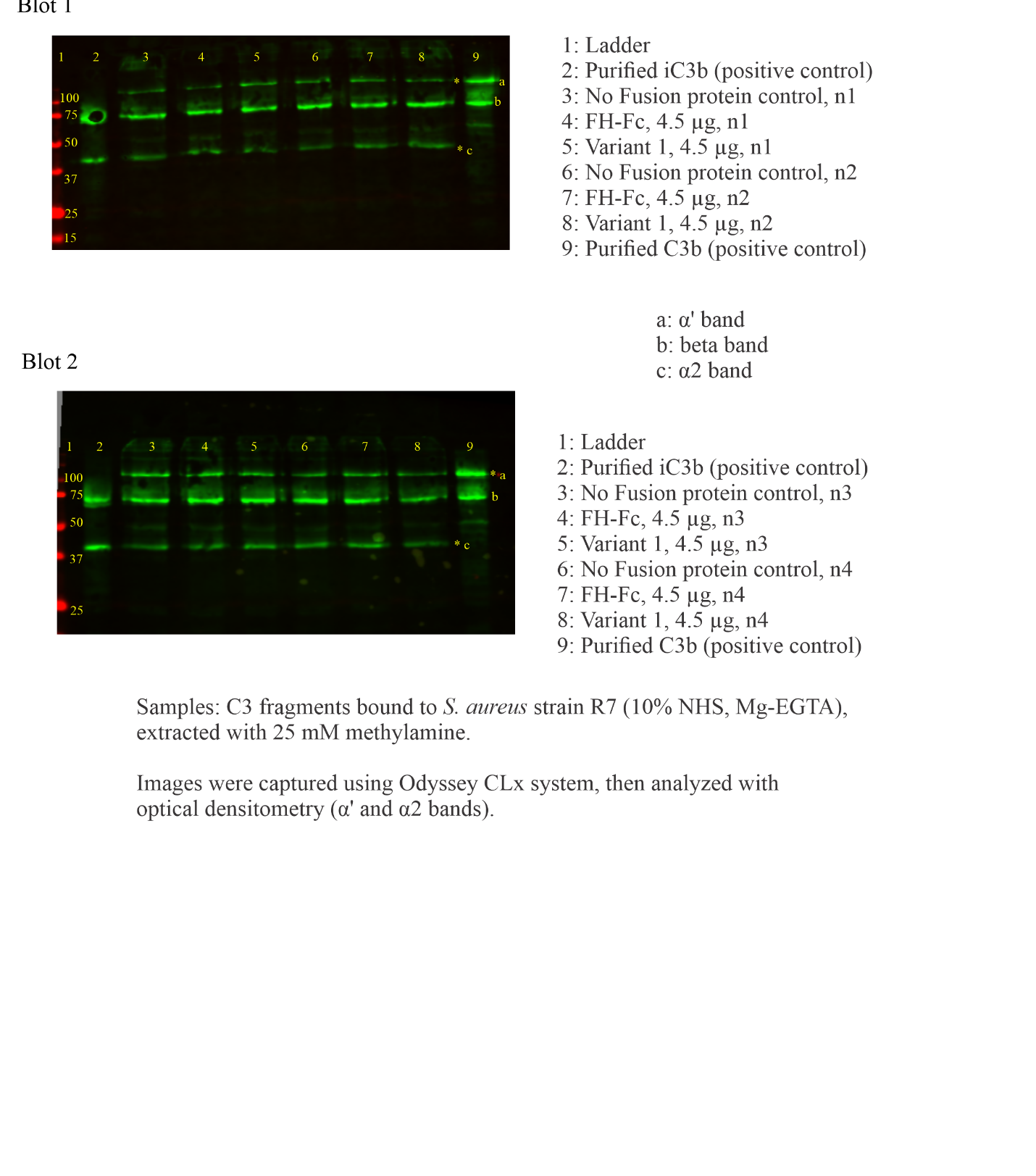


Table 2 Raw Data


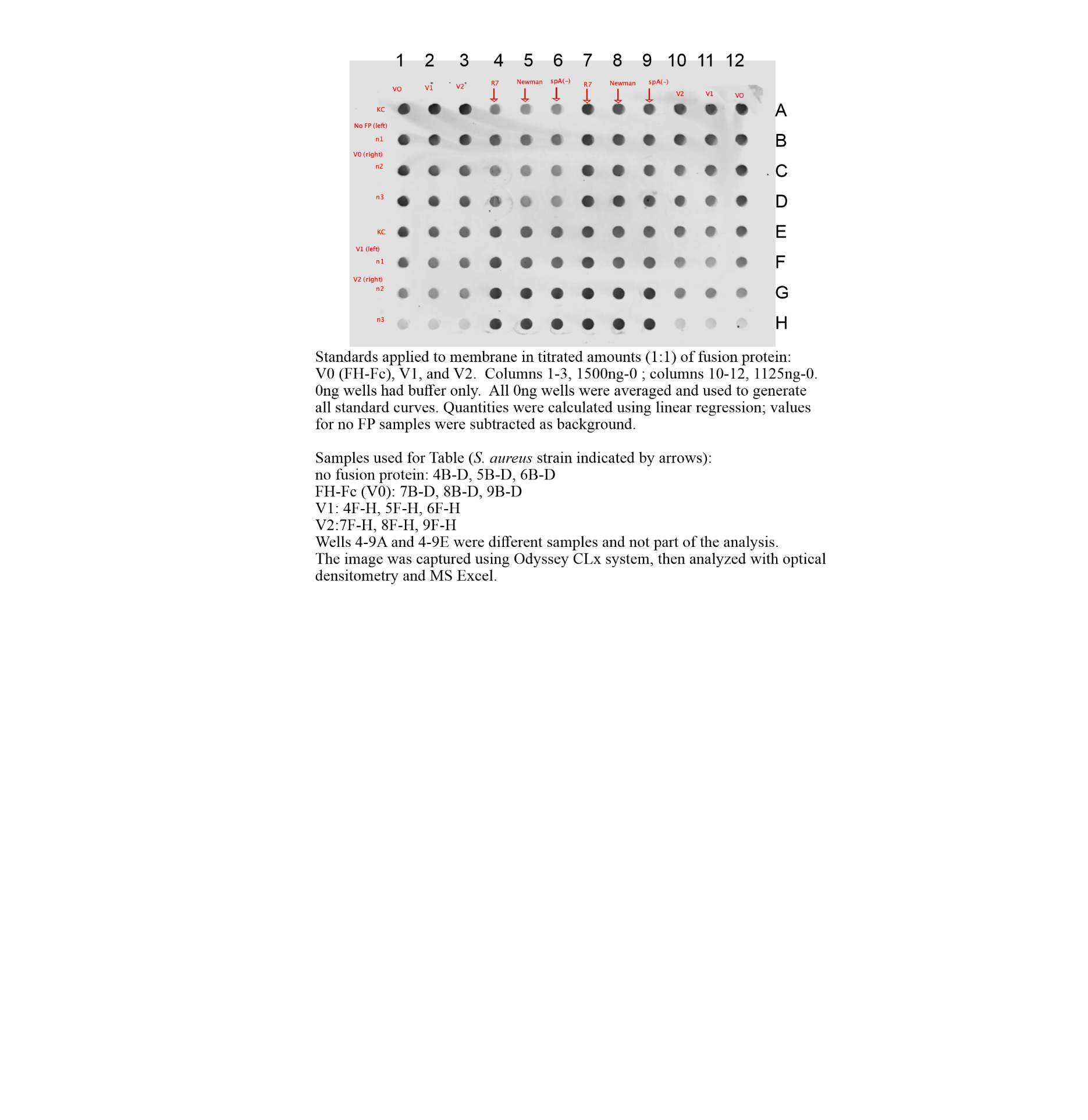
Table 4 Raw Data


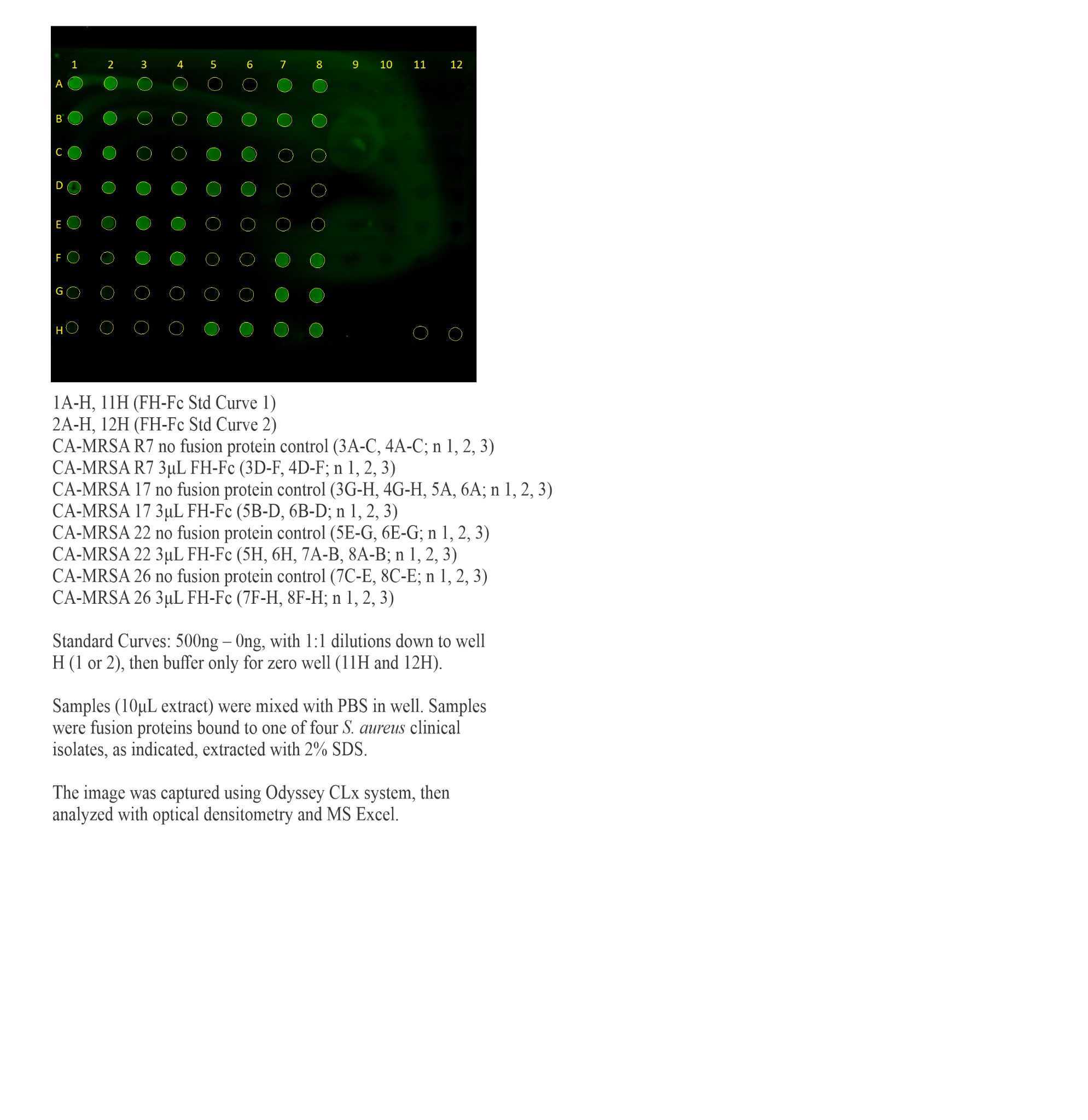

Supplement: S1 Raw images — (DOCX) [file pone.0265774.s002.docx]
